# Carbon Fibre-Reinforced Polymer Composites for Automotive Powertrain Components: A Comprehensive Review of Material Systems, Performance Requirements, and Functional Design Strategies

**DOI:** 10.3390/polym18141762

**Published:** 2026-07-18

**Authors:** Jozef Jaroslav Fekiač, Lucia Kakošová, Michal Krbata, Marcel Kohutiar, Alena Breznická, Pavol Mikuš, Maroš Eckert, Róbert Janík

**Affiliations:** 1 Faculty of Special Technology, Alexander Dubček University of Trenčín, Ku Kyselke 469, 911 06 Trenčín, Slovakia; lucia.kakosova@tnuni.sk (L.K.); michal.krbata@tnuni.sk (M.K.); marcel.kohutiar@tnuni.sk (M.K.); alena.breznicka@tnuni.sk (A.B.); pavol.mikus@tnuni.sk (P.M.); maros.eckert@tnuni.sk (M.E.); 2 Faculty of Industrial Technologies in Púchov, Alexander Dubček University of Trenčín, Ivana Krasku 491/30, 020 01 Púchov, Slovakia

**Keywords:** carbon fibre-reinforced polymer, CFRP, automotive powertrain, engine compartment, CF/PEEK, CF/PPS, CF/PEKK

## Abstract

Carbon fibre-reinforced polymer (CFRP) composites represent promising lightweight materials for automotive powertrain systems, where increasing demands for weight reduction, energy efficiency, and emission reduction are driving the replacement of conventional metallic components. However, automotive powertrain environments expose CFRP materials to elevated temperatures, cyclic mechanical loading, chemical exposure, and tribological interactions, creating complex degradation conditions that significantly influence long-term durability and reliability. This review systematically analyzes CFRP composites for automotive powertrain applications, focusing on the relationship between operational requirements, material selection, reinforcement architecture, manufacturing technologies, and degradation mechanisms. High-performance thermoplastic systems such as CF/PEEK, CF/PPS, and CF/PEKK are critically compared with conventional thermoset composites. CF/PEEK systems demonstrate superior thermomechanical stability, maintaining significant mechanical performance at temperatures approaching 250 °C and tensile strengths of approximately 1400–1600 MPa, whereas CF/PPS composites provide a more economically efficient compromise between thermal resistance, chemical stability, manufacturability, and recyclability for medium-temperature applications. The review further analyzes dominant degradation mechanisms, including creep deformation, fatigue damage, delamination, fibre–matrix interface degradation, and tribological wear. CFRP degradation is shown to result from the interaction of multiple coupled mechanisms rather than from isolated material failure modes. Tribological wear rates typically range from 10^−6^ to 10^−5^ mm^3^/(N·m), while creep–fatigue interactions may reduce component lifetime by up to 40–60% under combined thermomechanical loading. Advanced design strategies, including fibre orientation optimization, laminate architecture tailoring, thickness gradation, and hybrid metal–composite structures, are evaluated together with major manufacturing technologies such as injection moulding, compression moulding, overmoulding, automated fibre placement, and additive manufacturing. The presented review establishes an integrated framework linking material systems, operating conditions, manufacturing processes, and durability requirements for automotive powertrain applications. The analysis demonstrates that no universal CFRP system exists for all powertrain components and that optimal material selection requires balancing thermal stability, fatigue resistance, tribological performance, manufacturability, recyclability, and economic constraints according to the specific operating conditions of each component category.

## 1. Introduction

Carbon fibre-reinforced polymer (CFRP) composites represent a key class of advanced structural materials widely used in the aerospace, automotive, and energy industries due to their high specific strength, stiffness, and corrosion resistance. In the automotive sector, increasing demands for vehicle lightweighting, improved fuel efficiency, and compliance with stringent emission regulations are strongly driving the implementation of lightweight materials, with CFRP composites emerging as a promising alternative to conventional metallic systems. Othman et al. [1] reported that the application of CFRP materials enables significant vehicle weight reduction while maintaining high mechanical strength and structural safety. Similarly, Khatib et al. [2] highlighted the growing importance of CFRP composites as replacements for steel and aluminium alloys in modern automotive applications, particularly in areas requiring a combination of low weight and high strength. At the same time, Aldosari et al. [3] emphasized that the development of CFRP materials is closely associated with the principles of the circular economy and the increasing focus on recycling composite materials in the automotive industry.

Despite the widespread use of CFRP components in vehicle body structures, their implementation in automotive powertrain systems remains relatively limited. The powertrain environment represents one of the most demanding operational conditions within a vehicle, as it is characterized by the combined effects of elevated temperatures, chemically aggressive operating fluids, cyclic mechanical loading, and tribological interactions. Hamzat et al. [4] reported that the combined action of mechanical, thermal, and chemical loading leads to progressive matrix degradation, weakening of the interfacial region, and subsequent damage initiation in composite materials. These conditions impose complex and often contradictory requirements on material properties, including fatigue resistance, creep stability, thermal resistance, and wear resistance. De Leon and Sweat [5] further emphasized the importance of interfacial engineering between fibres and the matrix, as the quality of the fibre–matrix interface significantly affects the stability of mechanical properties under elevated temperatures and long-term service loading.

Unlike metallic materials, CFRP composites exhibit pronounced anisotropy of mechanical properties and complex failure mechanisms governed by the interaction between fibres, matrix, and the interfacial region. Yang et al. [6] demonstrated that transverse tensile and compressive loading leads to matrix microcracking, delamination, and progressive fibre–matrix debonding, significantly influencing the overall mechanical behaviour of composites. In addition, polymer matrices are highly sensitive to temperature and environmental conditions, which may substantially affect the long-term durability of the material. Moe et al. [7] emphasized that understanding progressive failure mechanisms and accurately modelling the mechanical behaviour of composite systems are essential prerequisites for their safe implementation in dynamically loaded structural applications. Therefore, the direct substitution of metallic powertrain components with CFRP composites is far from trivial and requires a comprehensive understanding of material systems, design strategies, and failure mechanisms.

Current research is focused on expanding the application potential of CFRP composites in powertrain systems through the development of high-performance thermoplastic matrices such as PEEK, PPS, and PEKK, as well as emerging concepts involving vitrimer-based composites. Hussain et al. [8] identified fatigue life enhancement under cyclic loading as one of the major challenges associated with fibre-reinforced composites intended for powertrain applications. Gabrion et al. [9] highlighted the favourable thermomechanical behaviour of high-temperature thermoplastic CFRP composites, which enables their application under elevated service temperatures. Furthermore, Alshammari et al. [10] and Yao et al. [11] emphasized that thermoplastic CFRP composites represent a promising direction of development due to their superior toughness, improved repairability, and recyclability compared to conventional thermoset systems.

Another emerging research direction is the development of vitrimer-based composites, which combine the mechanical performance of thermosets with repairability, reprocessability, and recyclability. These materials are considered a promising route toward sustainable CFRP systems for future automotive applications [12,13,14,15].

Despite the considerable progress reported in the current literature, a systematic framework linking performance requirements, material selection, structural design, and application-specific conditions within automotive powertrain systems is still lacking. Most available studies focus primarily on isolated aspects, such as material characterization or the design of specific components, without addressing the broader interrelationship between operational requirements, material systems, and structural performance.

The aim of this review article is therefore to provide a systematic and integrated overview of CFRP composites for automotive powertrain applications. The article first defines the key performance requirements arising from the operational environment, followed by an overview of relevant material systems, including thermoset, thermoplastic, and vitrimer-based composites. Subsequently, the functional categories of powertrain components are analyzed in relation to material selection and design strategies. The review further discusses dominant failure mechanisms and manufacturing technologies and concludes with an assessment of future trends involving electrification, multifunctional composites, and sustainable material development.

**Figure 1:**
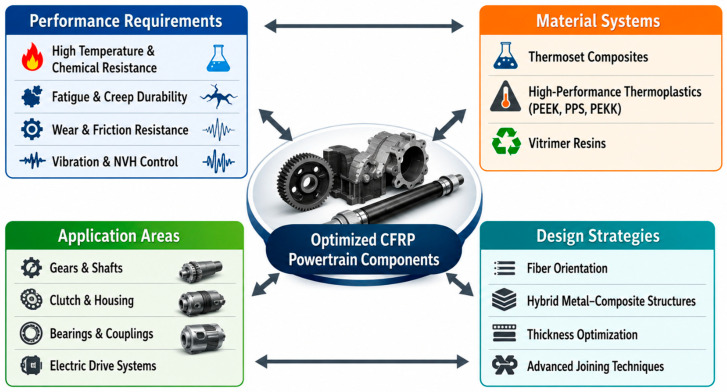
Conceptual framework linking performance requirements, material systems, applications, and design strategies of CFRP components.

In addition, this review establishes a systematic framework linking performance requirements, material systems, application areas, and design strategies of CFRP components in automotive powertrain systems, thereby providing a comprehensive basis for engineering decision-making in the design of these structures. Figure 1 presents the conceptual framework linking performance requirements, material systems, application areas, and design strategies of CFRP components in automotive powertrain applications.

## 2. Performance Requirements in Powertrain Systems

Before selecting an appropriate material system, it is essential to define in detail the operating conditions to which a powertrain component will be exposed throughout the entire service life of the vehicle. From a materials engineering perspective, the automotive powertrain represents an exceptionally complex operating environment in which elevated thermal loading, chemical exposure to operating fluids, cyclic mechanical loading, creep deformation, tribological interactions, and vibrational loading act simultaneously. The combined interaction of these factors frequently results in synergistic degradation mechanisms that significantly affect the long-term reliability and durability of components.

For this reason, the design of CFRP components must be based on a comprehensive evaluation of operating conditions, as individual performance requirements cannot be considered independently. Mechanical strength, thermal stability, chemical resistance, fatigue life, and tribological behaviour represent interrelated parameters that collectively determine the suitability of a material for a specific powertrain application. Typical automotive powertrain environments involve operating temperatures ranging from approximately 80–260 °C, cyclic loading conditions exceeding 10^6^–10^9^ cycles, continuous exposure to oils, fuels, and cooling fluids, and tribological contacts generating local contact stresses and wear rates in the range of 10^−6^–10^−5^ mm^3^/(N·m). These combined conditions impose strict requirements on the thermomechanical stability, fibre–matrix interface integrity, creep resistance, and long-term fatigue durability of CFRP systems.

**Figure 2:**
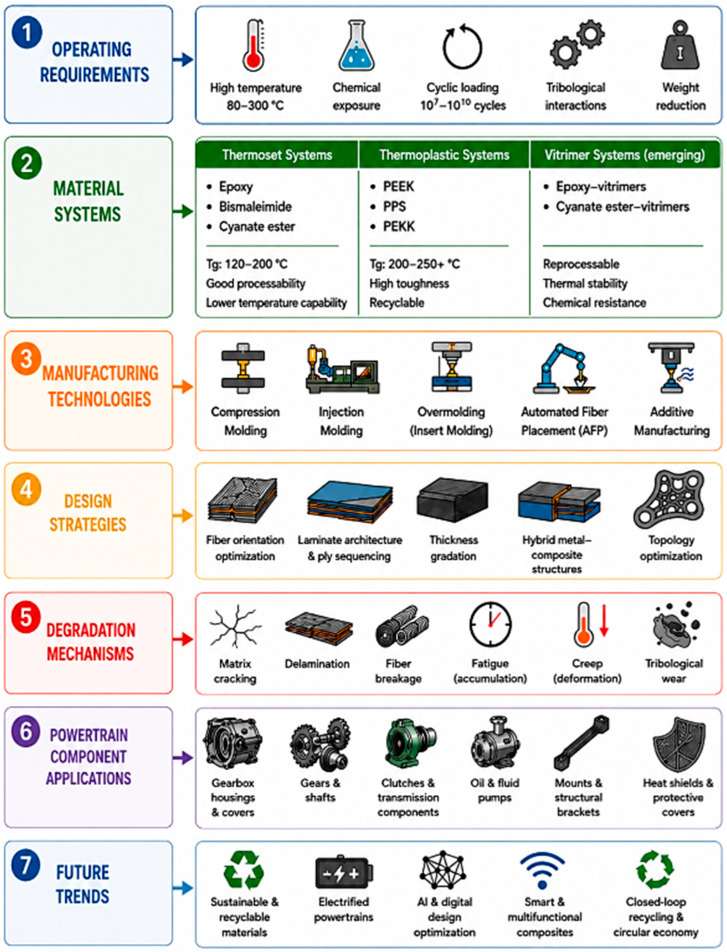
Integrated system-level framework for the implementation of CFRP composites in automotive powertrain systems, illustrating the interrelationship between operational requirements, material systems, manufacturing technologies, design strategies, degradation mechanisms.

Therefore, this section systematically analyzes the principal operational requirements acting on CFRP components in automotive powertrain systems and quantitatively relates them to critical material performance parameters, including thermal stability, fatigue resistance, tribological durability, and long-term structural reliability. Figure 2 summarizes the comprehensive system-level framework for the design and implementation of CFRP composites in automotive powertrain applications.

### 2.1. Thermal Loading Conditions

Thermal conditions within the engine compartment are highly heterogeneous and strongly dependent on the location of a specific component. In general, three temperature zones can be distinguished. The first zone includes components located in less thermally exposed regions of the engine compartment, such as covers, brackets, and supports, where long-term operating temperatures typically range from 80 to 120 °C, with short-term peaks not exceeding 150 °C [16]. The second zone comprises components in direct contact with oil or coolant, including oil pans, manifolds, and valve covers, where long-term temperatures range from 130 to 180 °C and peak temperatures may reach up to 200 °C [17]. The most demanding third zone corresponds to areas located near the exhaust system or turbocharger, where temperatures can temporarily reach 250–300 °C.

A key parameter for polymer matrix systems is the glass transition temperature (Tg), which must remain sufficiently higher than the maximum operating temperature to ensure stable mechanical performance and dimensional stability under service loading. In engineering practice, a safety margin of approximately 30–50 °C above the maximum operating temperature is generally required [18]. Consequently, the minimum required Tg is approximately 150–200 °C for the first temperature zone, 200–230 °C for the second zone, and above 280 °C for the third zone [19]. However, for semicrystalline thermoplastic matrices such as PEEK, PPS, and PEKK, thermal stability above Tg remains partially preserved due to the presence of crystalline regions that maintain structural integrity even after softening of the amorphous phase. As a result, these materials can operate at temperatures significantly above Tg, provided that sufficient stiffness and creep resistance are retained under long-term loading conditions [20].

**Figure 3:**
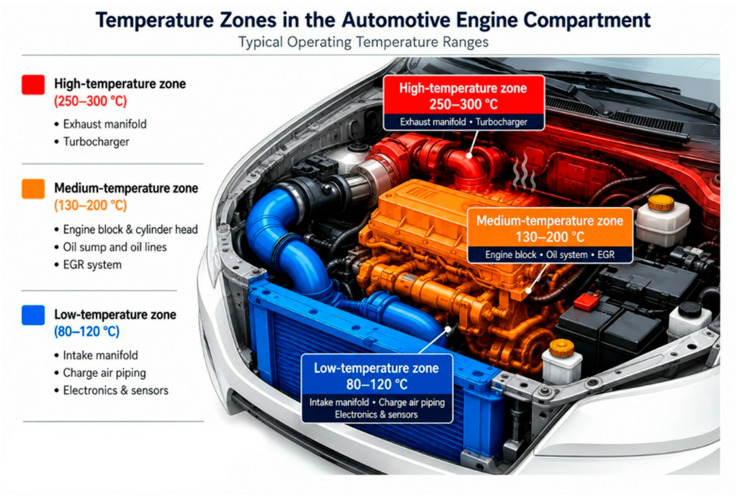
Temperature zones within the engine compartment with typical operating temperature ranges.

In addition to Tg, the thermal conductivity of the composite also plays an important role, particularly in terms of heat dissipation from thermally exposed regions. This effect can be significantly enhanced through the incorporation of graphene-based fillers [20] or carbon nanotubes [21]. Elevated temperatures simultaneously accelerate matrix plasticization, fibre–matrix interface degradation, creep deformation, and thermally induced residual stresses, all of which contribute to the progressive reduction of fatigue life and dimensional stability under cyclic operating conditions [21]. Figure 3 illustrates the division of the engine compartment into individual temperature zones together with the typical operating temperature ranges of selected powertrain components.

An additional critical factor is cyclic thermal loading, to which components are exposed throughout the entire service life of the vehicle. Repeated thermal cycles from ambient conditions (−40 to +40 °C) to maximum operating temperatures generate thermal stresses due to the mismatch between the coefficients of thermal expansion of carbon fibres (−0.5 to +1 × 10^−6^ °C^−1^) and polymer matrices (50–100 × 10^−6^ °C^−1^) [18]. This mismatch may lead to the initiation of matrix microcracks and progressive delamination under long-term cyclic loading conditions. Experimental studies further indicate that repeated thermomechanical cycling may reduce interlaminar shear strength and fatigue resistance by approximately 10–30%, depending on the laminate architecture, matrix system, and thermal exposure conditions [22].

These findings indicate that thermal resistance represents one of the principal limiting factors for the implementation of CFRP composites in automotive powertrain systems. Conventional epoxy matrices are generally restricted to low- and medium-temperature regions due to their limited long-term thermomechanical stability, whereas applications within the second and third temperature zones require high-performance thermoplastics such as PEEK, PPS, and PEKK or specialized high-temperature thermoset systems. A major challenge remains the trade-off between thermal resistance, manufacturability, processing temperature, and economic cost, which significantly influences the broader industrial implementation of CFRP composites in automotive manufacturing.

### 2.2. Chemical Exposure

Powertrain components are simultaneously exposed to multiple aggressive media, including engine oils (typically 5 W-30 or 0 W-20 at temperatures of 80–130 °C), coolant fluids (ethylene glycol–water mixtures with pH values of 6.5–8.5), transmission and differential oils (GL-5, with temperatures up to 150 °C), fuels (gasoline, diesel, and biofuels), and brake fluids (DOT 4, boiling point > 230 °C) [17]. The resistance of polymer matrices to these media strongly depends on their chemical structure [23]. Epoxy-based matrices exhibit increased fluid absorption and progressive degradation of fibre–matrix interfacial adhesion during long-term exposure to oils and elevated temperatures [24], whereas high-performance thermoplastics such as PPS and PEEK provide significantly superior chemical stability [25].

Experimental studies indicate that conventional epoxy matrices may absorb approximately 1–3 wt.% of fluids during long-term exposure, leading to reductions in glass transition temperature (Tg) of 10–25 °C and interlaminar shear strength (ILSS) losses of approximately 15–30% depending on exposure temperature and fluid composition [24]. In contrast, semicrystalline thermoplastics such as PPS and PEEK typically exhibit substantially lower fluid absorption (<0.5 wt.%), resulting in improved dimensional stability and reduced degradation of mechanical properties under chemically aggressive operating conditions [25].

Particular challenges are associated with modern biofuels and lubricant additives, which may accelerate matrix plasticization, hydrothermal ageing, and degradation of the fibre–matrix interface. Ethanol-containing fuels and ester-based additives are especially critical due to their ability to penetrate the polymer matrix and promote microcrack initiation during combined thermal and mechanical loading [23]. Elevated temperatures further accelerate diffusion processes and chemical ageing, contributing to progressive reductions in fatigue resistance and long-term structural reliability.

**Figure 4:**
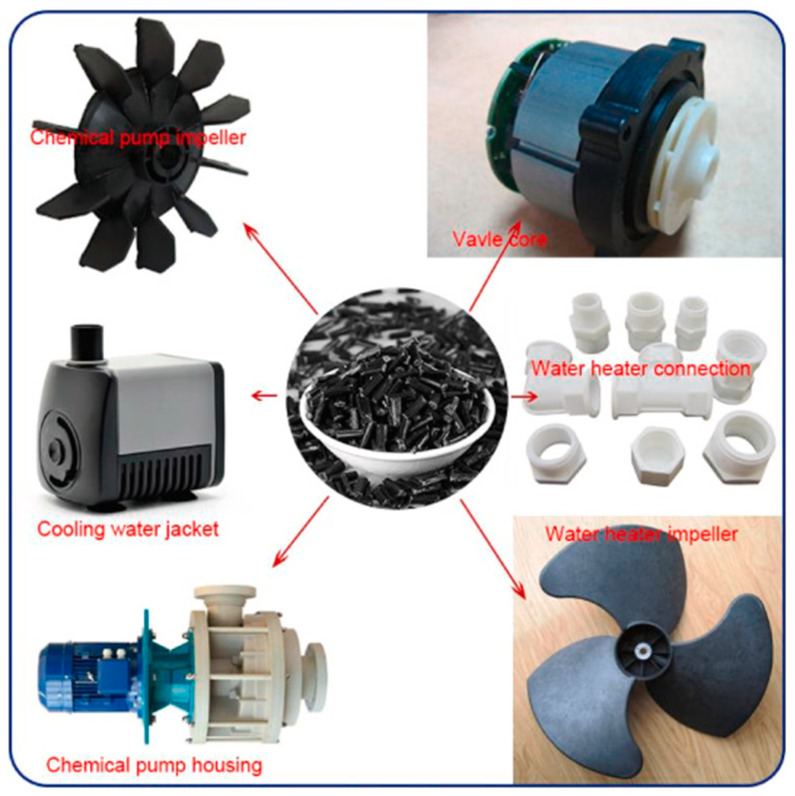
Examples of polymer and composite components used in environments exposed to chemicals and operating fluids [10].

Figure 4 illustrates representative powertrain components exposed to chemically aggressive and fluid-rich environments that impose increased demands on the chemical resistance of polymer matrix systems.

A quantitative indicator of chemical resistance is the fluid absorption after a defined exposure period. For engine compartment applications, an absorption value below 1 wt.% after 1000 h at operating temperature is generally considered acceptable [24]. Increased fluid absorption leads to matrix plasticization, reduction of the glass transition temperature (Tg), and a decrease in interlaminar shear strength (ILSS). In addition, it may initiate delamination processes at the fibre–matrix interface [23].

From the perspective of automotive powertrain applications, chemical resistance represents a critical factor particularly for components operating within the second temperature zone, where direct contact with operating fluids occurs. While thermoplastic systems such as CF/PPS and CF/PEEK exhibit excellent resistance to most aggressive media, epoxy-based systems remain limited under such conditions and often require additional modifications or protective coatings [10]. Typical fluid absorption values for CF/PPS and CF/PEEK systems remain below 0.5 wt.% even after prolonged exposure at elevated temperatures, resulting in significantly lower reductions in Tg and interlaminar mechanical properties compared with conventional epoxy-based systems. The long-term evaluation of coupled chemical, thermal, and mechanical degradation processes remains a major challenge, which may progressively reduce dimensional stability, fatigue resistance, and fibre–matrix interfacial integrity even under apparently acceptable absorption conditions [25].

### 2.3. Fatigue Loading

Powertrain components are subjected to cyclic mechanical loading throughout the entire service life of the vehicle. Drive shafts, gears, and connecting rods typically operate under high-cycle fatigue (HCF) conditions involving more than 10^7^ to 10^8^ loading cycles during vehicle operation [26]. Unlike metallic materials, for which a distinct fatigue limit can often be defined, polymer composites exhibit progressive stiffness degradation with increasing cycle number and do not possess a clearly defined fatigue limit. Experimental studies indicate that CFRP laminates may exhibit stiffness reductions of approximately 10–20% before final failure under high-cycle fatigue conditions, depending on stress ratio, temperature, and laminate architecture [27]. This behaviour significantly complicates the prediction of the long-term durability of CFRP components under service loading conditions [28].

**Figure 5:**
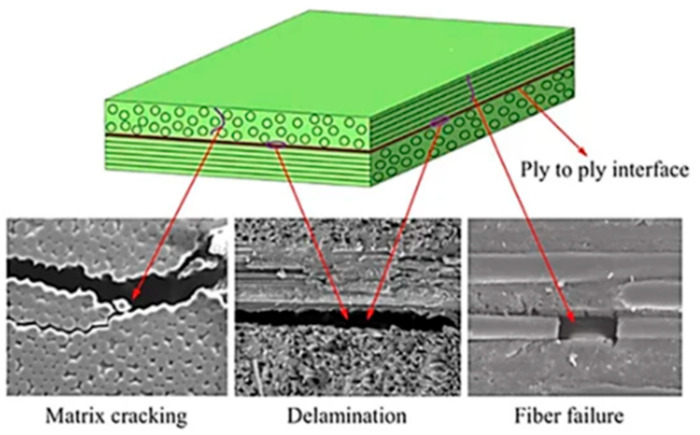
Failure mechanisms of CFRP composites including matrix cracking, delamination, and fibre fracture [14].

The fatigue process in CFRP composites generally proceeds through several interconnected stages: (i) initiation of matrix microcracks in stress concentration regions, (ii) crack propagation parallel to the fibres, (iii) fibre–matrix interfacial debonding, (iv) interlaminar delamination, and (v) final fibre fracture [28]. Figure 5 illustrates the principal stages of damage evolution in CFRP laminates, including matrix cracking, interlaminar delamination, and final fibre failure, which collectively contribute to the progressive degradation of mechanical performance under cyclic loading conditions.

The degradation rate depends on stress amplitude, temperature, loading frequency, humidity, and fibre orientation. A key characteristic describing fatigue behaviour is the S–N curve, which defines the relationship between stress amplitude and the number of cycles to failure, together with the threshold energy release rate required for crack propagation (GIth). Typical GIth values for CFRP laminates range approximately from 150 to 500 J/m^2^ depending on the matrix system, fibre architecture, and environmental conditions, with thermoplastic matrices generally exhibiting higher crack-growth resistance than conventional epoxy systems [29]. For CFRP drive shafts, resistance to loading at approximately 80% of the maximum stress for 10^6^ cycles without visible damage is typically required [21].

From the perspective of automotive powertrain applications, fatigue resistance represents one of the most critical limiting factors for the implementation of CFRP composites in dynamically loaded components. Unlike metallic materials, CFRP systems exhibit progressive damage accumulation and stiffness degradation without a clearly defined fatigue limit, which significantly complicates structural dimensioning and long-term durability prediction. A major challenge remains the reliable prediction of service life under combined thermomechanical loading and extremely high cycle counts exceeding 10^7^–10^9^ cycles, particularly for rotating components such as drive shafts and gears.

### 2.4. Creep Behaviour

Creep—defined as the time-dependent deformation of a material under prolonged static loading—represents a significant limiting factor for polymer matrices in automotive powertrain applications. Unlike metallic materials, where creep typically becomes relevant only above approximately 0.4 of the melting temperature, polymer matrices may exhibit creep behaviour even at substantially lower temperatures [30]. The creep deformation rate increases exponentially with both temperature and stress level [31]. In practical terms, this may lead to gradual dimensional instability of components, such as deformation of valve covers at fastening locations or changes in the geometry of bearing surfaces in composite transmission housings.

Creep behaviour is commonly quantified using the creep modulus E(t), which describes the reduction of effective stiffness over time, and the creep rate dε/dt during the secondary stage of the creep curve. For structural powertrain applications, a creep deformation below 0.5% after 1000 h at the maximum operating temperature and under stress corresponding to approximately 30% of the short-term material strength is typically required [30]. Semi-crystalline thermoplastic matrices such as PEEK, PPS, and PEKK exhibit significantly superior creep resistance compared to amorphous thermoplastics or epoxy-based matrices, making them particularly suitable for structurally loaded applications. Experimental studies indicate that CF/PEEK and CF/PPS systems may retain significantly higher long-term stiffness under elevated-temperature loading conditions, with creep deformation reductions exceeding 30–50% compared with conventional epoxy-based systems under equivalent thermomechanical conditions [31].

From the perspective of practical implementation, creep represents a critical issue especially for components exposed to prolonged static loading at elevated temperatures, including covers, flanges, and joint regions. Even relatively small deformations may result in loss of sealing performance or reduction of preload in bolted joints. The long-term stability of materials under combined thermal and chemical loading conditions remains a major challenge, which may further accelerate creep-related degradation processes. Particularly critical is the interaction between creep and cyclic fatigue loading, where time-dependent matrix deformation promotes interlaminar stress concentrations and accelerates the initiation and propagation of delamination. Under combined creep–fatigue conditions, component lifetime may decrease substantially compared with isolated fatigue loading, especially in thermally exposed joint regions and stress-concentration zones.

### 2.5. Tribological Conditions

Within automotive powertrain systems, two principal categories of tribological loading can be distinguished. The first category involves sliding contacts under liquid lubrication, such as seals, sliding bearings, and guide bushings, where the key parameters are the friction coefficient in the presence of lubricants and the surface resistance to abrasive wear. Under lubricated operating conditions, CFRP tribological systems typically exhibit friction coefficients in the range of approximately 0.05–0.15 depending on fibre orientation, matrix system, lubrication regime, and counterface material [32]. The second category comprises rolling and meshing contacts, typically represented by gears, where surface contact fatigue (pitting) and abrasive tooth wear dominate the degradation process [33].

In CFRP composite gears operating against steel counterfaces, two dominant wear mechanisms are generally identified: adhesive wear caused by localized microwelding of surface asperities and three-body abrasive wear, in which detached composite particles act as abrasive media within the contact zone [31]. The wear rate W (mm^3^/(N·m)) depends primarily on contact stress, sliding velocity, temperature, and lubrication conditions. For CFRP gears, the specific wear rate is typically reported within the range of 10^−6^ to 10^−5^ mm^3^/(N·m) at contact stresses between 60 and 150 MPa [31]. Compared with conventional metallic gear systems, CFRP composites generally provide lower density and improved vibration damping; however, their tribological performance remains strongly dependent on lubrication stability, contact temperature, and fibre–matrix interfacial integrity under repeated contact loading conditions [33].

**Figure 6:**
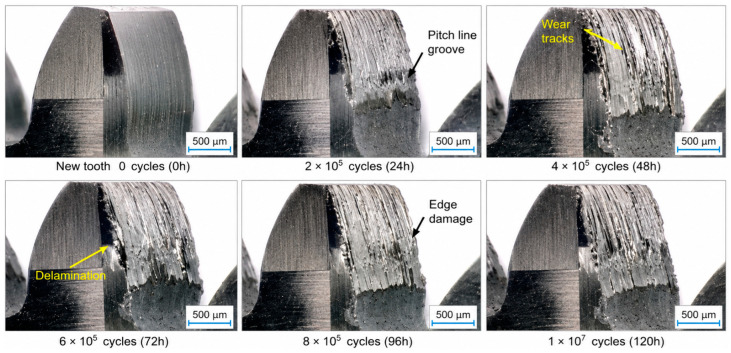
Evolution of damage in a CFRP component under cyclic loading, including wear formation, edge damage, and delamination [31].

Figure 6 illustrates the progressive degradation of a CFRP component under cyclic loading, characterized by the accumulation of damage in the form of wear, edge deterioration, and subsequent delamination.

From the perspective of automotive powertrain applications, tribological properties represent a significant challenge, particularly for components operating in direct contact with metallic counter-surfaces. Under wear conditions, exposed carbon fibres may emerge at the contact surface and act as abrasive particles, thereby accelerating the wear of the entire tribological system. Experimental studies indicate that CFRP components may exhibit substantially increased wear rates under insufficient lubrication or elevated contact temperatures, particularly in systems subjected to repeated rolling–sliding contact conditions.

Compared with conventional metallic systems, CFRP composites provide advantages in terms of reduced mass and improved vibration damping; however, their long-term tribological stability remains strongly dependent on fibre orientation, matrix toughness, lubrication regime, and fibre–matrix interfacial integrity. A major limitation remains the optimization of surface morphology and composite composition in order to minimize wear while maintaining the mechanical integrity and fatigue durability of the component under combined thermomechanical loading conditions.

### 2.6. Vibrational Loading and Damping

The automotive powertrain represents a source of broadband vibrational excitation, where the internal combustion engine generates harmonic excitations typically within the frequency range of approximately 10–500 Hz, while the gearbox and drive shafts transmit meshing frequencies and their harmonic components. The ability of a material to dissipate vibrational energy, commonly characterized by the loss factor tan δ, is significantly higher in polymer composites than in metallic materials [34]. This enhanced vibration damping capability directly contributes to noise reduction and improved acoustic comfort of the vehicle (NVH—noise, vibration, and harshness), while simultaneously reducing fatigue loading on surrounding components.

The damping behaviour of CFRP composites is strongly dependent on laminate architecture, fibre orientation, temperature, excitation frequency, and the quality of the fibre–matrix interface rather than being represented by a single material constant. Dynamic mechanical analysis (DMA) has shown that storage modulus decreases progressively with increasing temperature, while the loss factor (tan δ) increases as the polymer matrix approaches its glass transition region due to enhanced molecular mobility. Consequently, laminates designed primarily with 0° plies exhibit high longitudinal stiffness but relatively limited damping capacity, whereas ±45° plies increase shear deformation within the matrix, thereby enhancing energy dissipation under cyclic loading. Furthermore, improved fibre–matrix interfacial bonding contributes not only to higher static strength but also to more stable dynamic mechanical behaviour by reducing interfacial damage initiation during repeated loading cycles [9,33,35].

At the same time, sufficient dynamic stiffness must be maintained to ensure that the natural frequencies of components, such as drive shafts, do not coincide with the operational rotational range of the powertrain system. Otherwise, resonance phenomena may occur, leading to a significant increase in vibration amplitudes and accelerated material fatigue. In practical drivetrain design, the first natural frequency of rotating CFRP components is typically required to remain at least 20–30% above the maximum operational rotational frequency in order to avoid resonance-related amplification of vibration amplitudes and premature fatigue damage [26]. For this reason, the design of CFRP components requires modal analysis combined with fibre orientation optimization [26], enabling simultaneous optimization of torsional stiffness, dynamic stability, and vibration damping behaviour under operational loading conditions [23].

From the viewpoint of drivetrain engineering, vibration behaviour cannot be evaluated independently of component geometry and boundary conditions. Although maintaining the first natural frequency approximately 20–30% above the maximum operating rotational frequency represents a widely accepted preliminary design guideline, the actual safety margin depends on shaft length, diameter, wall thickness, laminate stacking sequence, bearing stiffness, joint configuration, and support conditions. Recent investigations of CFRP Double Cardan drive shafts demonstrated that modal analysis combined with harmonic response analysis is essential for identifying resonance-sensitive operating regions and optimizing laminate configurations. For example, numerical modal analyses reported natural frequencies exceeding 300 Hz for higher vibration modes, while harmonic response analysis identified critical excitation frequencies around 230 Hz that govern the maximum stress and deformation response. These findings demonstrate that reliable vibration design of CFRP powertrain components requires simultaneous optimization of stiffness, damping capability, laminate architecture, and modal characteristics rather than relying solely on static mechanical properties [36].

From the perspective of powertrain applications, vibrational behaviour represents a critical design trade-off between damping capability and structural stiffness. While high damping is advantageous in terms of NVH performance, insufficient stiffness may result in resonance effects and reduced component durability. Because automotive powertrain components operate under continuously changing environmental conditions, temperature-dependent dynamic behaviour must also be considered during fatigue design. Experimental investigations on CFRP automotive drive shafts have shown that variations in ambient temperature modify the mechanical behaviour of the polymer matrix and the fibre–matrix interface, leading to changes in torsional strength and fatigue life. Temperature-induced expansion or contraction of the resin matrix alters interfacial stress transfer and may reduce fatigue durability under variable service conditions. Therefore, vibration durability assessment should incorporate coupled thermo-mechanical loading together with realistic load spectra rather than considering vibration excitation under constant ambient conditions only [37]. From the perspective of powertrain applications, vibrational behaviour represents a complex interaction between laminate architecture, fibre orientation, operating temperature, excitation frequency, and structural boundary conditions. While high damping improves NVH performance and reduces dynamic stress amplitudes, excessive compliance may decrease critical rotational speed and increase susceptibility to resonance. Therefore, the design of CFRP powertrain components should integrate dynamic mechanical characterization, modal analysis, harmonic response analysis, and fibre orientation optimization to ensure adequate stiffness, vibration damping, and long-term fatigue durability under realistic service conditions. A summary of the most important operational and mechanical requirements imposed on CFRP composites in automotive powertrain environments is presented in Table 1.

Table 1 demonstrates that the performance requirements imposed on materials used in automotive powertrain systems are highly complex and strongly interrelated, making their simultaneous fulfillment a significant materials engineering challenge. In particular, the combination of high thermal resistance, low creep deformation, and superior fatigue durability requires optimization not only of the matrix system itself, but also of the reinforcement architecture and the quality of the fibre–matrix interface. In contrast to metallic materials, which generally exhibit relatively stable properties over a wide range of operating conditions, polymer composites must be specifically designed with respect to the intended application and service loading conditions. This complex set of requirements strongly influences the selection of suitable material systems and represents a key factor in the design of composite powertrain components.

Based on the defined performance requirements, individual material systems can be assigned to specific temperature zones within the engine compartment. For components operating in the first temperature zone (up to approximately 120 °C), CF/epoxy and low-temperature thermoplastic systems are generally suitable due to their favourable balance between performance and cost. The second temperature zone (130–200 °C) is dominated by high-performance thermoplastics, particularly CF/PPS and CF/PEKK, which combine good chemical resistance with sufficient thermal stability. For the most demanding conditions of the third temperature zone (above 200 °C), materials such as CF/PEEK or CF/BMI are required because they retain their mechanical properties even at elevated temperatures.

From a practical perspective, however, material selection is not governed solely by thermal loading conditions, but rather by the balance between mechanical performance, processability, and economic cost. Emerging vitrimer-based composites represent a promising alternative, particularly for applications requiring not only high mechanical performance but also recyclability and repairability. Nevertheless, their broader industrial implementation remains limited at the current stage of development.

## 3. Classes of Polymer Composites Used in Powertrain Systems

The definition of performance requirements presented in the previous section provides the foundation for a systematic overview of available material systems. Carbon fibre-reinforced polymer composites intended for automotive powertrain applications can generally be classified into four principal categories according to matrix type and reinforcement architecture: (i) thermoset composites, (ii) thermoplastic composites, (iii) emerging vitrimer-based systems, and (iv) composites with different fibre architectures. Each category offers a distinct balance between mechanical performance, thermal stability, processability, cost, and recyclability.

From an engineering perspective, material selection is strongly dependent on the operational temperature zone, loading mode, manufacturing strategy, and required service life of the component. Thermoset systems are typically utilized in highly loaded structural applications requiring low void content and high dimensional stability, whereas thermoplastic composites provide superior impact resistance, chemical stability, and recyclability. Continuous-fibre architectures are primarily used for rotating and structural components subjected to high cyclic loading, while short-fibre systems are generally preferred for injection-moulded components with complex geometries and lower structural demands.

Consequently, no universal CFRP system exists for all automotive powertrain applications. Instead, optimal material selection requires balancing thermal resistance, fatigue durability, tribological performance, manufacturing efficiency, and economic constraints according to the specific functional requirements and operating conditions of each component category.

### 3.1. Thermoset Composites

Thermoset matrices represent the traditional foundation of CFRP composites in structural applications. Their primary characteristic is the formation of a three-dimensional crosslinked polymer network during curing, which provides high stiffness, dimensional stability, and good adhesion to carbon fibres [25]. Owing to these properties, thermoset systems have long been used in applications requiring high mechanical performance [39]. The most widely used representatives are epoxy and bismaleimide (BMI) matrices. Epoxy systems dominate applications with moderate thermal requirements due to their favourable balance between cost and performance, whereas BMI matrices enable operation at higher service temperatures [39]. The principal limitations of thermosets include their inherent brittleness, limited resistance to prolonged exposure to elevated temperatures, and the inability to be recycled after curing, which has stimulated the development of alternative material systems.

#### 3.1.1. CF/Epoxy—Reference System

Carbon fibres embedded in epoxy matrices represent the most established and extensively studied CFRP system and serve as the reference benchmark for comparison with alternative composite materials. Epoxy matrices, most commonly based on diglycidyl ether of bisphenol A (DGEBA), form a three-dimensional crosslinked structure after curing, characterized by good adhesion to carbon fibres, low shrinkage, and stable mechanical properties [25]. Typical CF/epoxy laminates exhibit interlaminar shear strength values of 60–100 MPa, tensile strengths of 1000–1800 MPa, and elastic moduli of 120–180 GPa in the fibre direction [40].

The principal limiting factor of epoxy matrices in powertrain applications is the glass transition temperature (Tg), which for conventional formulations typically ranges between 120 and 150 °C [39]. Long-term exposure to elevated temperatures and oil-rich environments results in fluid absorption, matrix plasticization, and reduction of Tg, leading to decreased stiffness and dimensional stability of components [41]. Another important limitation is the relatively low fracture toughness (K_1_c ≈ 0.5–1.0 MPa·m^0.5^), together with the inability to repair or recycle cured thermoset structures [12,15].

For this reason, recent research has increasingly focused on the modification of epoxy matrices in order to improve their mechanical and functional performance. The incorporation of nanoparticles such as carbon nanotubes (CNTs), graphene nanoplatelets (GNPs), or graphene oxide (GO) has been shown to enhance interfacial adhesion, fracture toughness, and thermal conductivity [42]. The addition of polymeric toughening agents can significantly improve strength and crack propagation resistance without substantially reducing Tg [40]. Another effective strategy involves the use of thin-ply laminate architectures with ply thicknesses below 100 μm, which suppress the initiation and propagation of interlaminar cracks and improve the fatigue behaviour of composites [43]. Furthermore, it has been demonstrated that an appropriate combination of nanoparticles and optimized laminate architectures can substantially improve the overall resistance of composites to mechanical damage [44].

**Figure 7:**
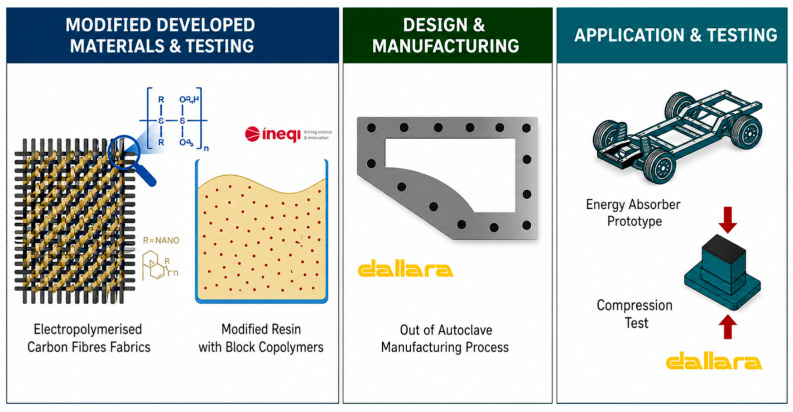
Process chain for the development of CFRP components including material modification, manufacturing, subsequent application, and testing [3].

Despite these advances, CF/epoxy systems remain fundamentally limited for high-temperature powertrain applications due to their restricted long-term thermomechanical stability and susceptibility to thermal and chemical ageing [39]. Typical applications include valve covers, protective housings, brackets, and secondary structural supports, where operating temperatures remain below approximately 120–150 °C and the primary design requirements involve moderate stiffness, dimensional stability, and weight reduction rather than extreme thermomechanical durability. An additional advantage is their compatibility with existing manufacturing infrastructure and their relatively low production cost compared to high-performance thermoplastic systems [45]. The integration of matrix modification strategies, laminate architecture optimization, manufacturing technologies, and performance evaluation methodologies relevant to CFRP powertrain components is schematically summarized in Figure 7.

#### 3.1.2. CF/Bismaleimide (BMI)—High-Temperature Thermoset Alternative

Bismaleimide (BMI) matrices represent a high-temperature thermoset alternative to conventional epoxy systems intended for more demanding applications. These polymers contain imide structures that, after curing, achieve glass transition temperatures in the range of 250–320 °C [46], significantly exceeding the capabilities of standard epoxy matrices [47]. CF/BMI composites typically exhibit tensile strengths of approximately 1200–1600 MPa while maintaining stable mechanical performance even at temperatures above 200 °C [46], making them suitable for applications within the second and third temperature zones of automotive powertrain systems.

The principal disadvantage of BMI matrices is their high brittleness, which is generally more pronounced than in epoxy-based systems, Typical fracture toughness values for BMI systems remain relatively low (K_1_c ≈ 0.4–0.8 MPa·m^0.5^), which increases their susceptibility to crack initiation and interlaminar damage under cyclic loading conditions [47]. These factors significantly limit their broader implementation in large-scale automotive production.

Within automotive powertrain applications, CF/BMI composites are particularly suitable for components exposed to elevated temperatures, such as regions located near turbochargers or exhaust systems, Representative applications include thermal shielding panels, turbocharger-adjacent covers, high-temperature structural brackets, and localized reinforcement regions subjected to prolonged thermal exposure exceeding approximately 200 °C [46]. Their utilization is therefore generally restricted to specialized applications in which thermal resistance requirements outweigh economic and manufacturing limitations [47].

**Figure 8:**
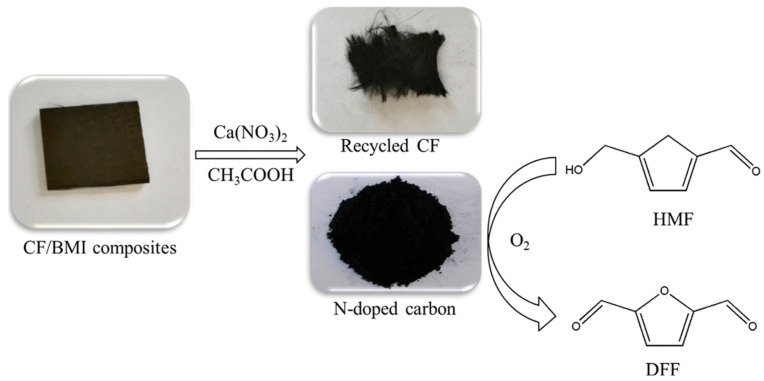
Recycling process of CFRP composites leading to the recovery of recycled carbon fibres and their subsequent processing [46].

In addition to their thermal performance, growing attention is being devoted to the end-of-life management and recyclability of high-temperature thermoset composites. Figure 8 illustrates the recycling pathway of CF/BMI composites together with the potential utilization routes of recovered carbon fibres and secondary carbonaceous products. The recycling of CF/BMI composites involves the chemical decomposition of the polymer matrix in order to separate carbon fibres from the thermoset structure. In the presented process, a combination of calcium nitrate Ca(NO_3_)_2_ and acetic acid CH_3_COOH is employed to degrade the BMI matrix and recover recycled carbon fibres. In addition to the fibres themselves, carbonaceous products in the form of nitrogen-doped carbon are also generated, which may subsequently be utilized in electrochemical or catalytic applications. The recycled products can therefore serve as feedstock for new composite systems or other value-added functional materials.

### 3.2. Thermoplastic Composites

Thermoplastic matrices represent a progressive alternative to thermoset systems, particularly for powertrain applications requiring improved thermal resistance, toughness, and recyclability. Unlike thermosets, thermoplastics do not contain a permanently crosslinked structure, allowing repeated melting and reprocessing without chemical degradation of the material [25]. Among the most important high-performance thermoplastics are materials from the polyaryletherketone (PAEK) family, such as PEEK and PEKK, together with PPS, which combine high thermal stability, chemical resistance, and favourable mechanical properties [48].

Compared with thermosets, thermoplastic systems generally exhibit superior impact resistance and improved behaviour under cyclic loading conditions. Thermoplastic CFRP systems typically exhibit significantly higher fracture toughness and impact resistance than conventional thermoset composites, with fracture toughness values frequently exceeding 1.5–2.5 MPa·m^0.5^ depending on matrix type and fibre architecture [48]. However, their processing is technologically more demanding and requires significantly higher processing temperatures. Typical processing temperatures range from approximately 300–400 °C for PEEK- and PEKK-based systems, substantially exceeding the processing conditions required for conventional thermoset composites [48]. These characteristics make thermoplastic CFRP composites suitable candidates for applications within the second and third temperature zones of automotive powertrain systems, where conventional epoxy-based materials reach their operational limitations. Continuous-fibre thermoplastic laminates are primarily utilized for highly loaded rotating and structural components, whereas short-fibre reinforced thermoplastics are more suitable for injection-moulded housings, covers, and fluid management components requiring high geometric complexity and large-scale manufacturability.

#### 3.2.1. CF/PEEK—High-Performance Thermoplastic

Polyether ether ketone (PEEK) belongs to the group of high-performance semi-crystalline thermoplastics within the polyaryletherketone (PAEK) family and combines excellent thermal stability, chemical resistance, and mechanical performance. Typical material characteristics include a glass transition temperature of approximately 143 °C, a melting temperature around 343 °C, and continuous service temperatures up to approximately 250 °C [25]. CF/PEEK composites with high fibre contents (60–66 wt.%) typically achieve tensile strengths of 1400–1600 MPa and elastic moduli of 120–140 GPa [48], while retaining approximately 70–80% of their mechanical properties even at elevated temperatures (e.g., 150 °C). Unlike amorphous thermoset systems, CF/PEEK composites retain structural integrity above Tg due to the presence of a stable crystalline phase, which continues to provide load-bearing capability even after partial softening of the amorphous regions. This semicrystalline behaviour enables CF/PEEK systems to maintain relatively high stiffness and creep resistance under prolonged thermomechanical loading conditions [25].

**Figure 9:**
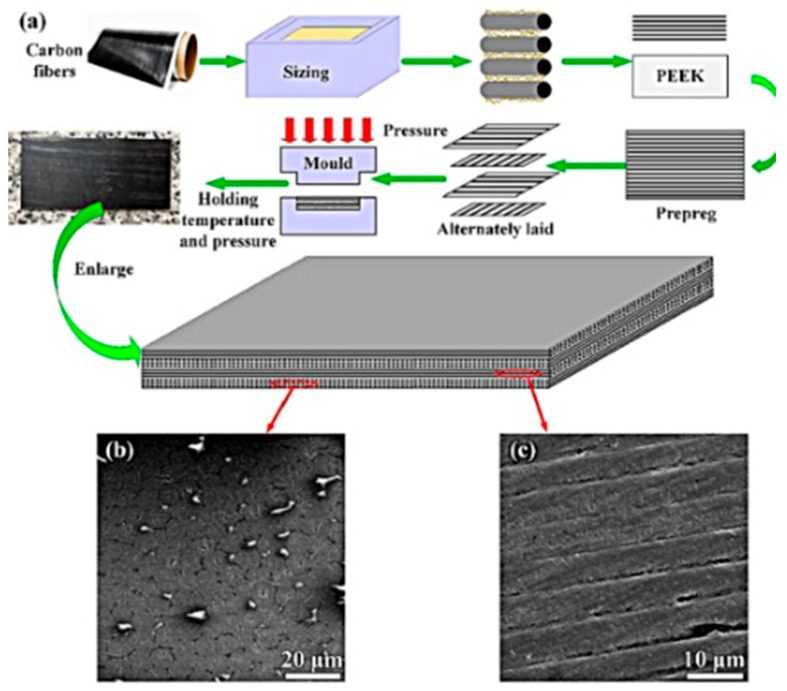
Manufacturing process of CF/PEEK composites including prepreg preparation, laminate stacking, and compression moulding together with the resulting microstructure: (**a**) schematic illustration of CF/PEEK laminate fabrication, (**b**) microstructure of the comp, (**c**) surface microstructure of the CF/PEEK laminate showing the aligned carbon fibres after compression moulding [48].

Figure 9 illustrates the manufacturing process of CF/PEEK composites, including prepreg preparation, laminate stacking, and compression moulding, together with the resulting material microstructure. The final mechanical performance of CF/PEEK composites strongly depends on the achieved degree of crystallinity, which is controlled by processing temperature, consolidation pressure, and cooling rate during manufacturing. Higher crystallinity generally improves stiffness, creep resistance, and thermal stability, whereas excessively rapid cooling may reduce crystallinity and increase residual stresses within the laminate structure [48].

A critical factor governing the mechanical behaviour of CF/PEEK composites is the quality of the fibre–matrix interface. PEEK is chemically inert, which complicates the use of conventional epoxy-based sizings [38]. Effective solutions include the application of thermoplastic sizings based on PEEK or PEI, as well as surface modification of carbon fibres using CNTs or plasma activation [31], resulting in increases of interlaminar shear strength (ILSS) typically in the range of approximately 15–35% depending on fibre treatment and processing conditions [49]. The manufacturing of CF/PEEK components is technologically more demanding than that of thermoset systems and requires processing temperatures in the range of 360–400 °C together with elevated consolidation pressures. Advanced technologies such as automated fibre placement (AFP) and prepreg compression moulding enable the production of components with very low void contents (<1%) and controlled microstructures [34,48].

An additional factor significantly influencing the final material properties is the degree of matrix crystallinity, which strongly depends on the cooling rate during processing [34]. Increased crystallinity improves elastic modulus and yield strength, but may simultaneously reduce material toughness. Typical crystallinity levels for processed CF/PEEK laminates range between approximately 25–40%, depending on cooling conditions and manufacturing route, with higher crystallinity generally associated with improved thermomechanical stability and creep resistance [37]. Owing to the combination of high thermal resistance, chemical stability, fatigue durability, and thermomechanical stability, CF/PEEK composites represent one of the most suitable material systems for highly loaded automotive powertrain components operating within the second and partially third temperature zones, particularly in applications where conventional epoxy-based systems cannot ensure long-term structural reliability.

#### 3.2.2. CF/PPS—Carbon Fibre Reinforced Polyphenylene Sulfide Composites for High-Temperature Powertrain Applications

**Figure 10:**
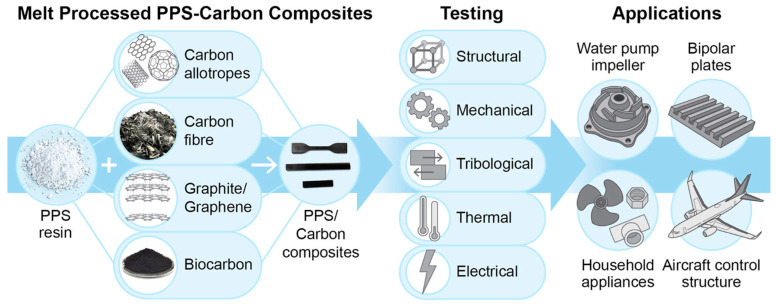
Schematic illustration of PPS carbon fibre composites showing the relationship between material composition, resulting properties, and application areas [30].

Polyphenylene sulfide (PPS) is a semi-crystalline thermoplastic characterized by an aromatic structure containing sulfur bridges, providing exceptional chemical resistance and good thermal stability. Continuous-fibre CF/PPS laminates typically achieve tensile strengths of approximately 700–1200 MPa and elastic moduli in the range of 70–140 GPa depending on fibre architecture, fibre volume fraction, and manufacturing route [21,22]. Short-fibre injection-moulded CF/PPS systems generally exhibit substantially lower stiffness and strength due to reduced effective fibre length and less efficient load transfer. PPS composites maintain mechanical integrity at temperatures up to approximately 240 °C (short-term exposure up to 270 °C) and exhibit inherent flame resistance without the need for halogen-based additives [21,22]. This behaviour is enabled by the semicrystalline structure of PPS, where crystalline domains preserve structural stability and creep resistance even above the glass transition temperature of the amorphous phase. Within automotive powertrain environments, PPS is particularly suitable for applications exposed to engine oils, fuels, and coolant fluids. The relationship between matrix composition, fibre architecture, resulting thermomechanical properties, and representative automotive powertrain applications of CF/PPS systems is summarized in Figure 10. Representative applications include oil pans, valve covers, transmission housings, fluid management components, and hybrid structural brackets operating within the second temperature zone of automotive powertrain systems.

Despite their advantageous properties, CF/PPS composites remain less extensively represented in the literature compared with CF/PEEK systems, primarily due to the historical research focus on PEEK-based materials. Nevertheless, experimental studies have demonstrated that short-fibre or injection-moulded CF/PPS systems typically achieve tensile strengths of approximately 700–900 MPa and elastic moduli around 30–40 GPa [23], whereas continuous-fibre laminates may exhibit substantially higher stiffness and strength depending on fibre architecture and fibre volume fraction. CF/PPS systems maintain good fatigue resistance even under high fractions of maximum applied stress, while the absorption of operating fluids generally remains below 1 wt.%, supporting their suitability for applications involving direct contact with aggressive media [21,23].

Although the glass transition temperature of PPS (Tg ≈ 85–100 °C) is lower than that of PEEK, Tg is not the principal limiting factor for semi-crystalline materials operating at elevated temperatures. The crystalline phase of PPS ensures structural stability up to temperatures approaching its melting point (~280 °C) [30,48]. One limiting factor of CF/PPS composites is the quality of the fibre–matrix interface, since PPS exhibits lower surface energy and poorer compatibility with conventional fibre surface treatments. To improve interfacial adhesion, plasma activation and fibre surface modification techniques are commonly employed [24], enabling sufficient interlaminar strength to be achieved [25].

Owing to the combination of chemical resistance, low fluid absorption, favourable processability, and lower manufacturing cost compared with CF/PEEK systems, CF/PPS composites represent one of the most economically viable material systems for medium-temperature automotive powertrain applications operating within the second temperature zone.

#### 3.2.3. CF/PEKK—Emerging Alternative with a Wider Processing Window

Polyether ketone ketone (PEKK) belongs to the polyaryletherketone (PAEK) family and represents a high-performance thermoplastic system combining elevated thermal stability, high mechanical performance, and improved processing flexibility compared with conventional CF/PEEK composites. Compared with PEEK, PEKK exhibits a higher glass transition temperature (Tg ≈ 160–165 °C) and a lower crystallization rate [26], enabling improved control of crystallinity during processing and reducing the risk of residual internal stresses. The slower crystallization kinetics of PEKK enable improved control of the final laminate microstructure and reduce the probability of non-uniform crystallinity distribution during manufacturing, which is particularly important for large or geometrically complex structural components [27]. CF/PEKK composites achieve mechanical properties comparable to CF/PEEK systems, with tensile strengths typically ranging from 1300 to 1500 MPa and elastic moduli of approximately 110–130 GPa in the fibre direction [26]. Owing to their higher Tg and favourable thermal stability, these composites maintain stable mechanical properties even at elevated temperatures, making them suitable for applications within the second temperature zone of automotive powertrain systems. Representative applications include thermally loaded structural housings, rotating drivetrain supports, hybrid overmoulded structures, and additively manufactured CFRP components requiring a combination of thermal stability, dimensional precision, and reduced residual stress formation.

A significant advantage of PEKK is its broader processing window, which enables processing under lower crystallization rates and provides additional time for laminate consolidation. This characteristic is particularly advantageous for manufacturing technologies such as automated fibre placement (AFP) and additive manufacturing [27], where precise control of the thermal cycle is critical for the quality of the final component [28]. On the other hand, slower crystallization may result in a higher fraction of amorphous phase if cooling conditions are not properly optimized, which may reduce stiffness or thermal resistance. Therefore, precise thermal management during processing represents a key factor for achieving optimal material performance [28].

CF/PEKK composites therefore represent an attractive material system for automotive powertrain applications requiring a combination of high thermal resistance, dimensional stability, and improved manufacturability. Their broader processing window and favourable crystallization behaviour make them particularly suitable for advanced manufacturing technologies such as AFP, automated consolidation processes, and high-performance additive manufacturing systems.

### 3.3. Vitrimer-Based CF Composites

Vitrimer-based composites represent an emerging class of polymer composite materials that combine selected advantages of conventional thermosets and thermoplastics. Unlike traditional thermoset matrices, which form permanent and irreversible crosslinked networks after curing, vitrimers contain dynamic covalent bonds capable of thermally activated exchange reactions. As a result, vitrimer matrices can retain thermoset-like dimensional stability and chemical resistance under normal service conditions while enabling repair, reshaping, reprocessing, and recycling at elevated temperatures [12,22]. From the perspective of automotive powertrain applications, this combination is particularly attractive because conventional CFRP thermosets provide high stiffness and fibre–matrix adhesion but remain difficult to recycle, whereas thermoplastics offer improved recyclability but often require high processing temperatures and more demanding consolidation conditions.

The most frequently investigated vitrimer systems for CFRP composites are epoxy-based vitrimers incorporating transesterification, boronic ester exchange, disulfide exchange, imine exchange, or other dynamic covalent mechanisms [12,15]. Among these, transesterification-based epoxy vitrimers are especially relevant because they can be designed using resin chemistries close to conventional epoxy systems while introducing thermally activated network rearrangement. This allows the matrix to dissipate stress, partially repair microdamage, and enable fibre recovery after chemical degradation. Therefore, vitrimer composites may offer a route toward structural CFRP materials that are not only mechanically efficient but also compatible with circular economy strategies.

Recent experimental studies demonstrate that vitrimer-based CFRP systems can achieve mechanical performance comparable to conventional epoxy-based composites. Lin et al. [13] developed a bio-based epoxy vitrimer reinforced with carbon fibres using vacuum resin infusion. The resulting CFRP composites achieved tensile and flexural strengths of approximately 543 MPa and 414 MPa, respectively, while maintaining degradability in ethylene glycol and allowing the recovery of carbon fibres without significant deterioration of their surface morphology. These results indicate that bio-based vitrimer matrices can provide a realistic compromise between structural performance and recyclability, although their long-term durability under combined thermal, chemical, and fatigue loading remains insufficiently validated for powertrain environments.

**Figure 11:**
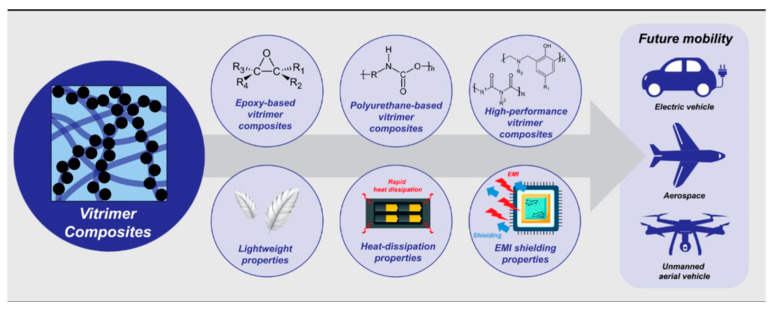
Overview of vitrimer composites including their properties and potential applications in mobility and aerospace sectors [22].

Figure 11 summarizes the main classes of vitrimer composites together with their multifunctional characteristics and potential application areas. Unlike conventional thermoset composites, vitrimer matrices can be designed using different polymer chemistries, including epoxy-, polyurethane-, and other high-performance systems, while maintaining dynamic covalent bond exchange that enables repairability and recyclability [12,22]. In addition to their structural lightweight nature, vitrimer composites may provide improved heat dissipation and electromagnetic interference (EMI) shielding, which are increasingly important for electrified powertrain systems and future mobility platforms. These multifunctional capabilities further broaden their potential beyond conventional structural applications, particularly in electric vehicles, aerospace structures, and unmanned aerial systems [14].

A particularly important aspect for powertrain components is the stability of the fibre–matrix interface, since fatigue damage, delamination, creep–fatigue interaction, and thermally induced microcracking often initiate in the interfacial region. Rahman et al. [43] demonstrated that tailoring the vitrimer–carbon fibre interface through dynamic covalent bonding can significantly improve interfacial adhesion. In their study, carbon fibre-reinforced vitrimers with diol-functionalized carbon fibres exhibited approximately 43% higher interfacial adhesion compared with composites containing pristine fibres. The same system achieved a tensile strength of approximately 731 MPa, which was reported to be 26% higher than that of unmodified vitrimer composites and 49% higher than that of the reference epoxy CFRP system. These findings suggest that vitrimer matrices should not be considered only as recyclable alternatives to thermosets, but also as platforms for active interfacial engineering in CFRP structures.

From a processing perspective, vitrimer composites are also relevant for scalable manufacturing technologies. Kumar et al. [14] emphasized that pultrusion combined with vitrimer matrices may represent a promising pathway for producing sustainable structural composite profiles. In conventional pultruded thermoset composites, the permanently crosslinked matrix prevents reshaping and makes recycling highly difficult. In contrast, vitrimer-based pultruded composites could potentially retain high fibre alignment and continuous-fibre reinforcement while enabling post-forming, repair, and recycling through bond exchange reactions. This processing route may be relevant for elongated automotive components, reinforcement profiles, brackets, shafts, and semi-structural elements where continuous fibre alignment is required.

For automotive powertrain applications, vitrimer composites should currently be regarded as a forward-looking material class rather than a directly validated replacement for CF/PEEK, CF/PPS, CF/PEKK, or CF/BMI systems. Their main potential lies in applications where high specific mechanical performance, improved fibre–matrix interface stability, repairability, and recyclability are required simultaneously. Possible future application areas include secondary powertrain covers, thermally exposed brackets, housings, vibration-damping structural elements, and hybrid metal–composite assemblies. However, for highly loaded rotating parts such as drive shafts, gears, or connecting rods, vitrimer CFRP systems still require extensive validation under high-cycle fatigue, creep–fatigue interaction, elevated temperature exposure, oil and coolant ageing, and tribological loading.

A key limitation of vitrimer systems is that most currently available experimental studies focus on material-level properties, recyclability, repairability, and interfacial adhesion rather than on full-scale automotive powertrain components. Therefore, validated data on long-term fatigue behaviour, creep resistance above 120–180 °C, dimensional stability in oil-rich environments, and performance under combined thermomechanical loading remain limited. In addition, the bond exchange mechanisms that enable repair and reprocessing may also influence stress relaxation, creep deformation, and long-term stiffness retention at elevated temperatures. These effects must be carefully quantified before vitrimer CFRP composites can be considered for safety-critical or highly loaded powertrain applications.

Overall, vitrimer-based CFRP composites represent a promising emerging direction in the development of sustainable high-performance composites. Compared with conventional epoxy systems, they offer clear advantages in repairability, reprocessability, recyclability, and potential closed-loop recovery of carbon fibres. Compared with high-performance thermoplastics, they may offer lower processing viscosity and better compatibility with established liquid composite moulding technologies. Nevertheless, their implementation in automotive powertrain systems remains at an early stage. Future research should focus on standardized testing under powertrain-relevant conditions, including thermal ageing, fluid exposure, high-cycle fatigue, creep–fatigue coupling, interlaminar fracture toughness, and component-level validation. Until such data become available, vitrimer composites should be positioned as a highly promising but still emerging material class rather than as a fully validated substitute for established thermoplastic or high-temperature thermoset CFRP systems.

### 3.4. Fibre Architecture—Short, Long, and Continuous Fibres

In addition to matrix type, reinforcement architecture has a major influence on the mechanical properties, anisotropy, and processability of composites. For automotive powertrain applications, three principal categories can be distinguished: composites reinforced with short, long, and continuous fibres. Short-fibre composites (SF-CFRP, fibre length 0.1–1 mm) are primarily intended for injection moulding of geometrically complex components. The random fibre orientation results in approximately isotropic behaviour, although with significantly lower mechanical performance, where tensile strength typically ranges between 150 and 300 MPa [13]. In powertrain systems, these materials are mainly used for covers, brackets, and other components in which geometric complexity dominates over structural loading requirements [23].

Long-fibre composites (LFT-CFRP, fibre length 5–25 mm) represent a compromise between processability and mechanical performance. Short- and long-fibre systems provide superior manufacturability and shorter production cycles suitable for large-scale automotive production, whereas continuous-fibre laminates require more advanced manufacturing routes such as prepreg processing, AFP, or compression moulding in order to achieve optimal fibre alignment and low void content. These materials are most commonly manufactured by compression moulding or injection moulding of preforms and achieve tensile strengths of approximately 400–700 MPa [23], making them suitable for semi-structural components with moderate geometric complexity.

Composites reinforced with continuous fibres (CF-CFRP, fibre length comparable to the dimensions of the component) enable full utilization of the mechanical potential of carbon fibres, with tensile strengths of 1000–1800 MPa and elastic moduli of 120–180 GPa [23]. Consequently, they are essential for the most highly loaded powertrain components, such as drive shafts, connecting rods, and gears [39]. A key design parameter in continuous-fibre composites is fibre orientation and laminate stacking sequence. Laminates with (±45°) sequences are particularly suitable for torsion-loaded components, (0°/90°) configurations improve bending stiffness, and multidirectional stacking provides quasi-isotropic behaviour appropriate for complex loading conditions. Fibre architecture additionally exerts a strong influence on fatigue resistance, crack propagation behaviour, and damage tolerance. Continuous-fibre laminates with optimized stacking sequences generally exhibit superior fatigue durability and lower stiffness degradation under cyclic loading compared with short-fibre systems, where stress concentrations around fibre ends frequently accelerate crack initiation and interfacial debonding [23,24,25,26].

The selection of fibre architecture therefore represents one of the key design decisions in CFRP powertrain engineering, directly influencing mechanical performance, fatigue durability, manufacturing complexity, and production economics. While short-fibre systems enable efficient manufacturing of geometrically complex components with reduced production costs, continuous-fibre laminates remain indispensable for highly loaded structural and rotating components requiring maximum stiffness, strength, and long-term durability. The principal characteristics of the most important CFRP material systems applicable to automotive powertrain applications are summarized in Table 2.

The comparison of individual material systems demonstrates that no universal solution exists for all automotive powertrain applications. Consequently, optimal material selection must be based on the combined evaluation of operating temperature, mechanical loading mode, fatigue requirements, chemical exposure, manufacturing constraints, and economic considerations associated with the specific component category. Thermoset systems such as CF/epoxy and CF/BMI offer excellent mechanical properties and structural stability; however, their major disadvantages include limited recyclability and lower processing flexibility. In contrast, high-performance thermoplastics—particularly CF/PEEK, CF/PPS, and CF/PEKK—provide a combination of chemical resistance, thermal stability, and recyclability, although their implementation is often limited by higher cost and technological complexity of manufacturing. CF/PEEK currently represents the reference high-performance thermoplastic system for thermally loaded automotive powertrain applications, whereas CF/PPS and CF/PEKK provide more economically viable alternatives balancing thermal stability, manufacturability, and production cost. Vitrimer composites represent an emerging material class combining recyclability and repairability with mechanical properties approaching those of conventional thermoset systems; however, their broader industrial implementation still requires validation of long-term thermomechanical stability, fatigue durability, and large-scale manufacturing feasibility.

## 4. Functional Categories of Powertrain Components

Composite powertrain components can be systematically classified according to their primary mechanical function into five principal categories. Such a functional classification enables a more comprehensive understanding of the relationship between operational loading conditions, required material properties, and the suitability of specific CFRP systems for a given application. Unlike classifications based solely on vehicle type or material system, this approach provides a more integrated perspective on the design of composite components under realistic service conditions.

The individual categories include components predominantly subjected to torsional loading, bending, contact stresses, thermal and chemical exposure, as well as applications requiring high dimensional stability and vibration damping capability. Components operating within the first temperature zone are generally limited by stiffness, dimensional stability, and manufacturing cost, whereas applications within the second and third temperature zones are increasingly governed by thermomechanical stability, creep resistance, tribological durability, and resistance to combined thermal–chemical degradation processes. Each category imposes different requirements on mechanical performance, thermal resistance, creep behaviour, fatigue durability, and tribological properties. While some applications demand maximum specific strength and stiffness at minimum weight, others are primarily limited by long-term stability at elevated temperatures or resistance to aggressive operating fluids. Highly loaded rotating and structural components typically require continuous-fibre laminates optimized for torsional or bending stiffness, whereas geometrically complex housings and auxiliary components are more commonly manufactured using short- or long-fibre reinforced thermoplastic systems compatible with injection or compression moulding technologies.

**Figure 12:**
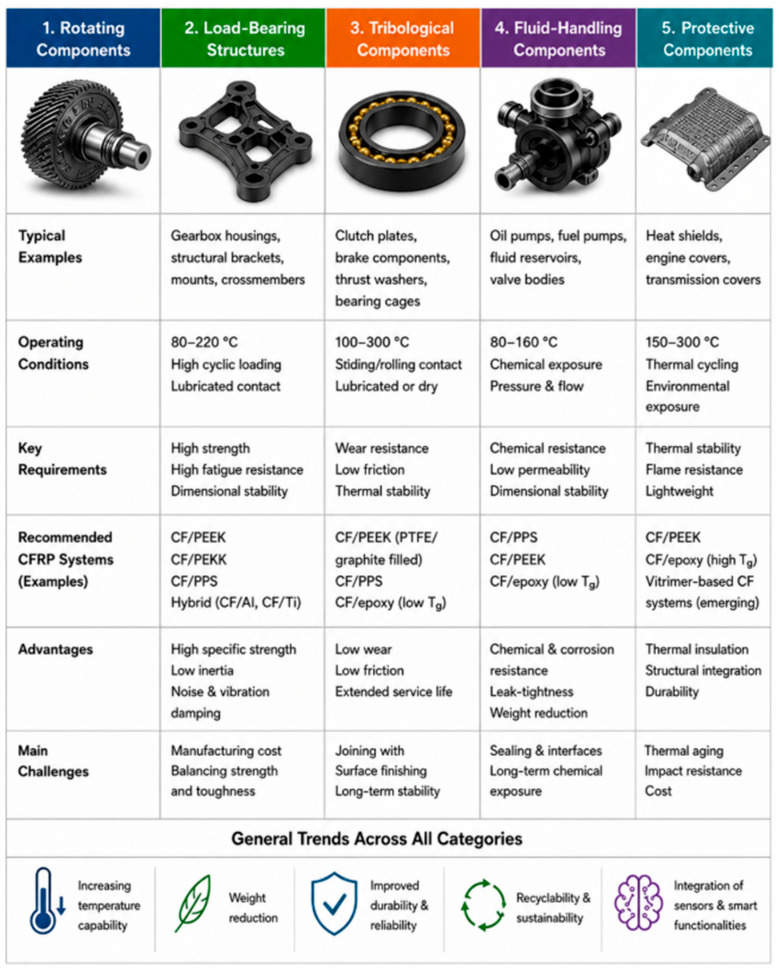
Classification of CFRP composite applications in automotive powertrain systems, including operating conditions, key performance requirements, recommended material systems, advantages, and main technological challenges associated with rotating, load-bearing components.

This functional classification establishes a direct engineering linkage between operational loading conditions, material selection, reinforcement architecture, and manufacturing strategy, thereby forming the basis for integrated design and decision-making in CFRP automotive powertrain development. Furthermore, the functional classification facilitates the identification of applications in which CFRP composites can effectively replace conventional metallic materials, as well as areas where their implementation remains limited by technological or economic constraints. CFRP composites can be implemented across various categories of automotive powertrain components, although individual applications differ significantly in terms of operating conditions, loading mechanisms, and material performance requirements. Figure 12 summarizes the principal application areas, typical service conditions, recommended CFRP material systems, as well as the main advantages and limiting factors associated with each category of components.

### 4.1. Load-Bearing Components

Load-bearing powertrain components, particularly connecting rods and crankshafts, represent the most mechanically critical elements of the entire engine system. Connecting rods are subjected to alternating tensile and compressive loading generated by the combined effects of combustion pressures and piston inertia forces, with stress amplitudes typically reaching approximately 300–600 MPa at frequencies corresponding to engine rotational speed [15]. Over the service life of an engine, this results in extremely high cycle counts on the order of 10^8^–10^9^ cycles [50], imposing exceptionally demanding requirements on the high-cycle fatigue resistance of the material.

Crankshafts are simultaneously subjected to combined bending, torsional loading, and localized contact stresses within bearing regions, where a critical design aspect is the elimination of torsional resonances and the maintenance of sufficient dynamic stiffness of the system [15]. In addition to fatigue resistance, dimensional stability under thermal loading is also essential for these components, since even small deformations may affect bearing functionality and the precision of the mechanical system.

For such applications, continuous-fibre CFRP composites—particularly CF/epoxy and CF/PEEK systems—appear to be the most suitable solutions because they enable efficient load transfer along the fibre direction. The optimal fibre orientation is predominantly aligned with the principal loading direction (0°), while supplementary ±45° plies provide improved resistance to shear and torsional loading [15]. Properly optimized CFRP laminate architectures may enable weight reductions of approximately 60–80% compared with steel counterparts, resulting in reduced inertial loading, improved dynamic response, and lower loading of bearing assemblies and adjacent drivetrain components [15].

Despite these advantages, the implementation of CFRP materials in load-bearing components remains limited by the orthotropic nature of their mechanical behaviour. The material exhibits significantly higher strength and stiffness in the fibre direction than in the transverse direction, requiring precise analysis of loading conditions and careful optimization of laminate stacking sequences for specific applications. Particularly critical regions include bolt and pin holes, where fibre continuity is interrupted and stress concentrations are generated [50], potentially leading to premature fatigue damage. Consequently, the design of joints and structural details represents one of the key factors governing the reliable implementation of CFRP composites in load-bearing powertrain components.

**Figure 13:**
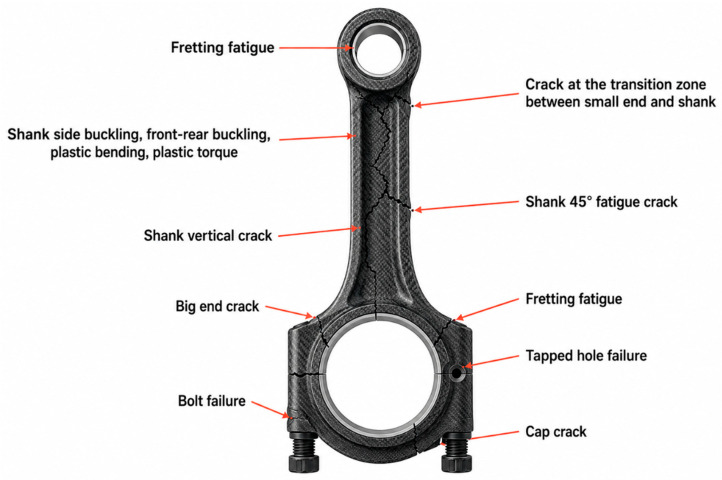
Localization of typical connecting rod failures under cyclic loading, including fatigue crack initiation and joint damage [15].

Dimensional stability under thermal loading is equally important, since temperature fluctuations directly influence clearances in bearings and piston pin assemblies. Figure 13 illustrates the localization of critical stress concentration regions and representative fatigue failure mechanisms in CFRP connecting rods subjected to high-cycle loading conditions.

### 4.2. Sliding and Tribological Components

Sliding components, such as bearings, seals, and guide bushings, are primarily defined by requirements for low friction coefficient, high wear resistance, and chemical stability in oil-rich environments [40]. Their operation involves hydrodynamic, mixed, and boundary lubrication regimes, with insufficient lubrication conditions representing the most critical operating state. For such applications, CF/PEEK and CF/PPS composites are most commonly employed due to their favourable combination of tribological performance and chemical resistance. Under oil-lubricated conditions, CFRP tribological systems typically exhibit friction coefficients in the range of approximately 0.05–0.10 depending on fibre orientation, matrix system, lubrication regime, and contact pressure [51].

**Figure 14:**
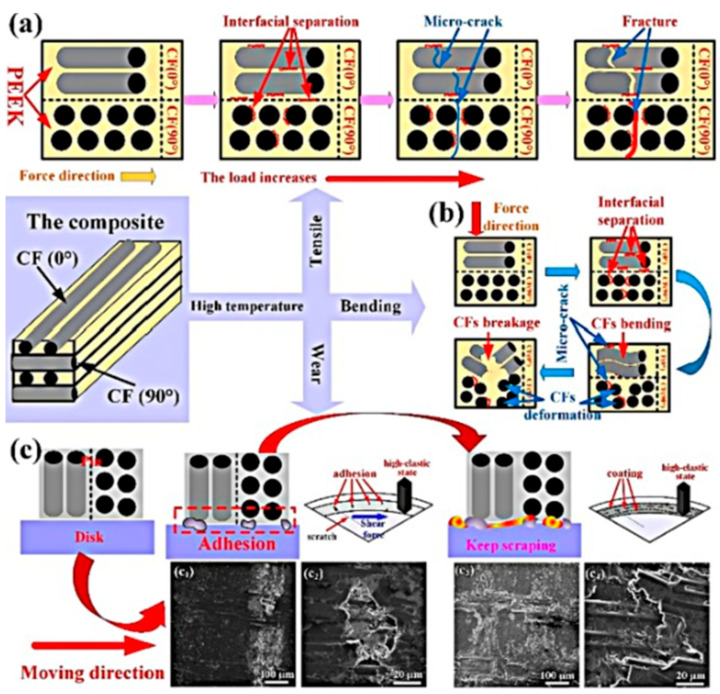
Complex degradation mechanisms of CFRP composites under combined mechanical and tribological loading: (**a**) initiation and propagation of interfacial damage and microcracks under tensile loading, (**b**) deformation and fracture of carbon fibres under bending, (**c_1_**) initial adhesive interaction between the CFRP composite and the counterface; (**c_2_**) development of adhesive wear accompanied by matrix removal and fibre exposure; (**c_3_**) continued sliding resulting in progressive material removal and accumulation of wear debris; (**c_4_**) formation of a transfer layer and severe surface damage after prolonged sliding wear [40].

A particularly critical problem of sliding CFRP components is the wear of carbon fibres at the contact interface. During abrasive wear processes, fibres fracture into short fragments that remain trapped within the contact zone and act as abrasive particles, thereby accelerating wear of the opposing surface. This effect is especially pronounced in contact with soft aluminium alloys, where hard carbon fibres may induce severe abrasive damage [40]. Figure 14 illustrates representative tribological degradation mechanisms of CFRP composites under combined mechanical, thermal, and sliding-contact loading conditions, including fibre fragmentation, abrasive wear, and interlaminar damage progression.

Another important factor is the anisotropy of wear behaviour depending on fibre orientation relative to the sliding direction. Anti-parallel (AP) fibre orientation relative to the sliding direction generally results in higher wear rates and increased fibre fragmentation compared with parallel (P) and normal (N) orientations, where more stable tribological behaviour is typically observed [51]. From an application perspective, the tribological behaviour of CFRP composites therefore represents a complex problem governed by the interaction between material properties, fibre orientation, and operating conditions. A major engineering challenge remains the simultaneous minimization of counter-surface wear, stabilization of friction behaviour, and maintenance of long-term tribological durability under combined thermal, mechanical, and lubrication-dependent operating conditions.

### 4.3. Rotating Components

Rotating components such as drive shafts and gears are subjected to combined torsional, bending, and dynamic loading conditions [52]. During operation, high torque transmission occurs simultaneously with cyclic loading and vibrational excitation, imposing demanding requirements on fatigue resistance, dynamic stiffness, and material stability [53]. A key parameter of rotating systems is the critical rotational speed, which must remain sufficiently above the maximum operating speed in order to avoid resonance and excessive vibration amplitudes [52]. In practical drivetrain applications, the first critical rotational speed is typically designed to remain at least 20–30% above the maximum operational speed in order to prevent resonance-induced amplification of dynamic stresses and premature fatigue damage [52].

Due to their low density and high elastic modulus, CFRP shafts provide significantly higher natural frequencies together with superior vibration damping capability compared with metallic counterparts [52]. Reduction of rotating mass decreases rotational inertia, significantly improving transient drivetrain response, reducing rotational inertia effects, and decreasing loading of bearing assemblies and adjacent rotating components. An additional advantage of CFRP materials is their ability to effectively damp vibrations and noise, thereby improving the NVH (noise, vibration, harshness) characteristics of the vehicle.

The mechanical behaviour of rotating CFRP components strongly depends on fibre orientation and laminate stacking sequence. Optimal configurations generally include combinations of ±45° plies providing high torsional stiffness, 0° plies carrying axial loads, and 90° plies improving circumferential stability of the component [52]. Proper combinations of these orientations enable optimization of the balance between strength, stiffness, and dynamic performance [53]. The suppression of delamination under cyclic torsional loading remains a critical design consideration, since interlaminar failure may lead to progressive stiffness degradation and eventual structural failure.

For gear applications, hybrid constructions combining steel contact surfaces with CFRP supporting structures are most commonly employed [41]. Such solutions enable substantial weight reduction while maintaining sufficient contact durability of the gear teeth. Hybrid CFRP–steel gear concepts may enable weight reductions of approximately 30–60% compared with fully metallic systems while preserving the local contact durability required in highly stressed tooth-contact regions [54]. The principal limitation of CFRP materials remains their lower contact strength compared with hardened steel [41], together with sensitivity to localized contact stresses and abrasive wear [54]. During long-term operation, degradation of surface layers, delamination, or matrix damage may occur in the tooth root region.

Another major challenge in the design of rotating CFRP components is ensuring reliable joining between composite and metallic parts. Joint regions act as stress concentrators where fatigue crack initiation or interlaminar failure may occur. Consequently, the implementation of CFRP materials in rotating components requires a combination of optimized laminate architecture, precise dynamic design, and detailed analysis of service loading conditions.

### 4.4. Housing and Protective Components

Engine covers, transmission housings, and protective components are primarily subjected to assembly loads, internal fluid pressures, and repeated thermal cycling [55]. Although these components are not primary structural elements of the powertrain system, their function is critically important for overall system reliability, since they ensure protection of internal mechanisms, sealing integrity, and stability of mounting assemblies. In addition to mechanical loading, these components are exposed to vibrations, chemical action of oils and coolants, and localized thermal gradients [56].

Key requirements include sufficient stiffness, creep resistance in joint regions, and long-term dimensional stability at elevated temperatures [55]. Particularly critical regions include bolted joints and sealing flanges, where stress relaxation and loss of preload may occur as a consequence of creep behaviour of the polymer matrix. Even relatively small deformations may result in sealing failure or fluid leakage within the system.

For housing and protective components, composites reinforced with short or long carbon fibres are most commonly employed, enabling the production of geometrically complex parts using injection moulding or compression moulding technologies. A major advantage of CFRP-based solutions is the possibility of integrating reinforcing ribs, mounting features, and functional details directly into a single component, thereby reducing the number of individual parts and assembly operations. At the same time, weight reductions of approximately 30–50% compared with conventional metallic housings can be achieved while maintaining sufficient stiffness, dimensional stability, and assembly integrity under operational loading conditions.

An additional important aspect is the vibrational behaviour of housing structures. Due to the higher internal damping capability of polymer composites, CFRP housings can reduce the transmission of vibrations and noise more effectively than metallic components, thereby positively affecting the NVH characteristics of the vehicle. At the same time, sufficient dynamic stiffness must be maintained in order to avoid resonance phenomena or excessive deformation under service loading conditions.

**Figure 15:**
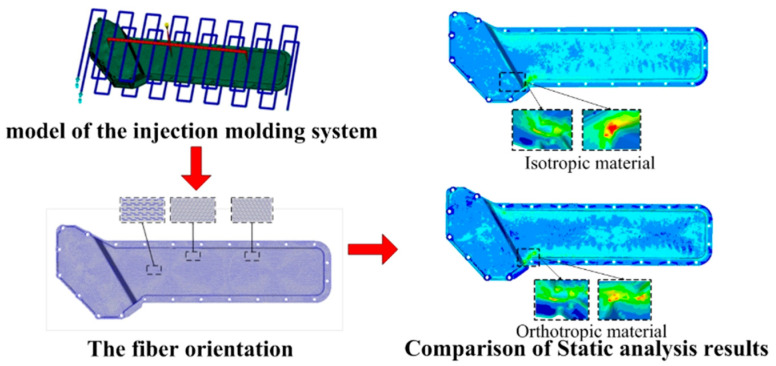
Influence of fibre orientation on the numerical analysis results of a composite component when comparing isotropic and orthotropic modelling approaches [55].

Figure 15 illustrates the influence of fibre orientation and material anisotropy on the numerical stress distribution and deformation behaviour of CFRP housing components, highlighting the differences between isotropic and orthotropic modelling approaches.

For these applications, short-fibre and long-fibre thermoplastic composites such as CF/PA66, CF/PPS, and CF/PEEK are most commonly employed [55]. A significant advantage of these material systems is the possibility of manufacturing geometrically complex components using injection moulding technologies, which enable the integration of mounting features, reinforcing ribs, and functional details directly into a single part [55]. In addition, thermoplastic CFRP composites provide low fluid absorption, favourable chemical stability in oil-rich environments, reduced component weight, and improved vibration damping behaviour compared with conventional metallic housing structures, which are particularly advantageous for automotive powertrain applications [56].

**Figure 16:**
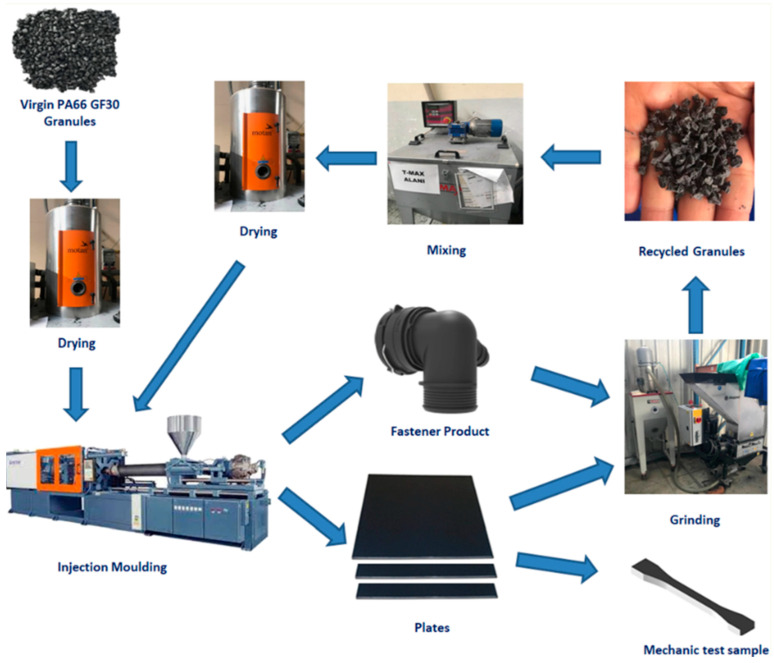
Schematic illustration of the closed-loop polymer processing cycle including injection moulding, recycling, and reprocessing stages [56].

The recycling approaches illustrated in Figure 7 are complemented by the practical processing cycle presented in Figure 16. However, dimensional stability and deformation caused by material shrinkage and anisotropy remain critical challenges [55]. Non-uniform fibre orientation during processing may lead to localized distortions that may significantly reduce assembly precision, sealing reliability, and long-term dimensional stability of the component under cyclic thermomechanical loading conditions [56].

### 4.5. Sealing and Fluid-Handling Components

Fluid-handling components such as intake manifolds, oil pans, cooling circuits, and fuel system components are subjected to combined chemical exposure, internal pressure, and thermal cycling [55]. During operation, these parts experience prolonged contact with engine oils, fuels, coolants, and their vapours, imposing demanding requirements on the chemical stability of the polymer matrix and the long-term dimensional stability of the component [23]. The primary requirement is the maintenance of sealing integrity throughout the entire service life of the vehicle, even under repeated fluctuations in temperature and pressure.

For these applications, CF/PA66 and CF/PPS composites are most commonly employed. PPS-based materials exhibit significantly lower moisture absorption and improved dimensional stability compared with polyamide systems [23], which is particularly advantageous for components exposed to long-term contact with operating fluids. CFRP-based solutions also enable the integration of complex internal channels, reinforcing ribs, and mounting features directly into a single component [55], thereby reducing the number of joints and potential leakage locations. An additional advantage is the reduction of component weight while maintaining sufficient structural stiffness.

A critical factor in the design of these components is the long-term creep behaviour of the material in bolted joints and sealing regions. At elevated temperatures, gradual stress relaxation and loss of preload may occur, leading to reduced sealing contact pressure, progressive leakage formation, and potential reduction of long-term system reliability under cyclic thermomechanical operating conditions [23]. This issue is particularly significant for components subjected to repeated thermal cycling, where creep deformation is combined with differences in thermal expansion between metallic and composite parts.

Another major challenge is ensuring long-term chemical stability in contact with modern fuels and operating fluids containing bioethanol, biodiesel fractions, oxidation products, and various performance-enhancing additives, which may accelerate matrix plasticization and fibre–matrix interfacial degradation processes. Fluid absorption may result in matrix plasticization, reduced stiffness, and progressive degradation of the fibre–matrix interface. For this reason, reliable implementation of CFRP materials in fluid-handling applications requires integrated optimization of matrix chemistry, fibre architecture, joint design, sealing geometry, and long-term resistance to coupled thermal–chemical degradation processes throughout the entire service life of the vehicle. A systematic classification of CFRP powertrain components according to dominant function, loading conditions, and suitable material systems is summarized in Table 3.

The comparative frameworks and decision-oriented tables presented in this review were developed through a structured synthesis of the published literature. The classifications, recommended material systems, manufacturing strategies, degradation mechanisms, and design guidelines were established by critically evaluating the reported mechanical performance, thermal stability, durability, processing characteristics, and application-specific requirements of CFRP composites for automotive powertrain systems. Where quantitative values are presented, they represent typical ranges reported across multiple experimental studies rather than results obtained under identical testing conditions.

The overview of component categories demonstrates that the potential of CFRP composites in automotive powertrain systems strongly depends on the specific application and the dominant loading mechanism. While load-bearing and rotating components are primarily governed by mechanical properties, fatigue durability, and dynamic behaviour, housing and fluid-handling components are mainly limited by chemical resistance, sealing capability, and dimensional stability. At present, the greatest implementation potential of CFRP composites lies in secondary, semi-structural, and moderately loaded components operating predominantly within the first and second temperature zones, where the combination of reduced weight, sufficient thermomechanical stability, and favourable manufacturability provides clear advantages over conventional metallic solutions. In contrast, for critically loaded components such as gears or high-pressure systems, their implementation remains limited mainly by contact strength, fatigue durability, and the reliability of composite-to-metal joints.

The selection of both material system and structural design therefore represents a multidimensional engineering optimization problem involving the simultaneous balancing of mechanical performance, fatigue durability, thermal and chemical stability, manufacturing feasibility, recyclability, and economic cost, highlighting the necessity of an integrated design framework for future CFRP automotive powertrain components.

## 5. Design Strategies for Functional Composite Powertrain Components

The selection of a suitable material system represents a necessary but not sufficient condition for the successful implementation of CFRP components in automotive powertrain systems. A key role is played by the design strategy, which includes fibre orientation, thickness gradation, geometric detail design, and integration of composites with metallic elements. These factors fundamentally influence mechanical properties, fatigue life, and the dynamic behaviour of the component. The optimal design strategy strongly depends on the functional category of the component, loading mode, operating temperature zone, and manufacturing technology, requiring simultaneous consideration of the performance requirements discussed in Section 2, Section 3 and Section 4.

This section summarizes the principal design approaches employed in the development of functional CFRP powertrain components, with emphasis on their influence on stiffness optimization, fatigue durability, dynamic performance, manufacturability, and long-term structural reliability under realistic automotive powertrain operating conditions.

### 5.1. Fibre Orientation

**Figure 17:**
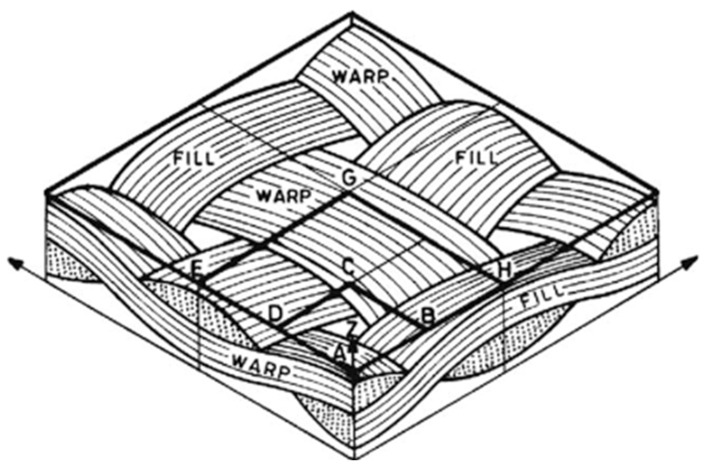
Schematic illustration of woven composite reinforcement showing fibre orientation in the warp and fill directions [2].

Fibre orientation is the most important design parameter of continuous CFRP composites because it directly determines the spatial distribution of mechanical properties within the component. Carbon fibres exhibit significantly higher elastic modulus (230–800 GPa) and tensile strength (3500–7000 MPa) in the axial direction than in the transverse direction [33], enabling targeted tailoring of stiffness and strength according to specific loading conditions [57]. However, the final structural performance of CFRP components is governed not only by the intrinsic properties of carbon fibres, but also by laminate architecture, fibre volume fraction, ply stacking sequence, and the quality of the fibre–matrix interface. Consequently, fibre orientation must be considered at the laminate and component levels rather than solely at the level of individual fibres. Figure 17 illustrates representative woven reinforcement architectures used in CFRP laminates together with their influence on load transfer mechanisms, in-plane stiffness, and anisotropic mechanical behaviour.

For powertrain drive shafts, torsional loading represents the dominant loading condition, corresponding to an optimal fibre orientation of ±45° relative to the shaft axis. This orientation maximizes shear stiffness and torsional strength [33], while additional 0° and 90° plies are incorporated to ensure sufficient bending stiffness and to increase the critical rotational speed [39]. Numerical optimization of the laminate stacking sequence of a CFRP shaft for a Formula-class racing vehicle demonstrated that the stacking sequence [90°/0°/−45°/+45°]_s_ provides the best combination of torsional stiffness, bending natural frequency, and low weight. Although this example originates from a high-performance motorsport application, the underlying design principles regarding fibre orientation, torsional stiffness optimization, and natural frequency control remain directly applicable to automotive drivetrain components operating under similar torsional loading conditions [33].

For gears, the situation is more complex, since meshing loads act in the direction normal to the tooth flank, while the composite gear must simultaneously withstand bending stresses at the tooth root and contact stresses at the tooth surface [32]. Woven laminate architectures consisting of multiple plies oriented at different angles provide improved quasi-isotropic resistance of the gear rim [31], whereas fibres within the hub and web regions may be predominantly radially oriented in order to maximize axial stiffness [32].

For connecting rods, the optimal orientation is typically 0° aligned with the principal loading axis, supplemented by ±45° plies to provide resistance against transverse loading and torsional stiffness under inclined loading conditions [37]. Modern design tools—particularly topology optimization and finite element-based fibre orientation optimization—enable automated determination of optimal laminate configurations for complex multi-criteria loading scenarios [57]. These tools are especially valuable in the design of components requiring simultaneous optimization of stiffness, fatigue resistance, and natural frequencies, parameters that are difficult to optimize concurrently using conventional iterative approaches.

Despite the availability of advanced numerical tools, the design of optimal fibre orientation remains a complex task, particularly for components subjected to multiaxial and dynamic loading conditions. A major engineering challenge remains the integration of numerical optimization methods with manufacturing constraints such as fibre steering limitations, minimum bending radii, ply drop-off requirements, and achievable laminate consolidation quality, all of which may restrict the theoretically optimal fibre architecture.

### 5.2. Thickness Design and Cross-Sectional Gradation

Thickness gradation—the intentional variation of ply number and laminate thickness along the component—represents an effective strategy for optimizing the weight and mechanical performance of CFRP structures. The principle is based on concentrating material in regions subjected to high stress while reducing thickness in less loaded areas, thereby minimizing weight while maintaining the required strength and stiffness [31,57].

For load-bearing components such as connecting rods and crankshafts, it has been demonstrated that graded cross-sections with localized reinforcement in critical regions (e.g., around pin holes or at locations of maximum bending moment) enable weight reductions of approximately 15–25% while maintaining sufficient fatigue safety [37]. In the case of housing and protective components, thickness gradation allows reinforcement of bolt connection regions and stress concentrators while simultaneously reducing the weight of large surface areas [31].

A particularly advanced approach involves the use of thin-ply laminates with ply thicknesses below 100 μm, which suppress the initiation and propagation of interlaminar cracks by reducing local stress concentrations [31,58]. This strategy is especially relevant for components subjected to cyclic fatigue loading, where it can significantly extend service life without increasing structural weight. In addition to improving fatigue performance, thickness gradation enables more uniform stress distribution within the laminate, thereby reducing the likelihood of localized delamination and premature failure in highly loaded regions.

From an application perspective, thickness gradation represents an effective tool for optimizing the performance of CFRP components; however, its implementation is limited by manufacturing constraints and the need for precise laminate quality control. For complex geometries and large-scale production, its practical utilization remains technologically demanding.

### 5.3. Ribbing and Geometrical Stiffening Features

Ribbing—the integration of reinforcing ribs into component geometry—represents an effective method for increasing the bending stiffness of thin-walled structures without significantly increasing weight. In CFRP components, this approach is particularly important because composite walls are generally thinner than their metallic counterparts and would otherwise be susceptible to excessive deformation under service loading conditions [59].

The design of ribs in CFRP composites is closely related to the manufacturing process. In technologies such as injection moulding, rib geometry strongly influences the local orientation of short fibres and consequently the resulting mechanical properties. Melt flow simulation is therefore essential for predicting fibre orientation and optimizing the structural design. For engine covers, T-shaped ribs with heights approximately two to three times the wall thickness have been shown to provide an effective compromise between stiffness and weight, whereas excessively high ribs may lead to local defects and material inhomogeneity. Experimental and numerical studies have shown that appropriately designed rib structures may increase local bending stiffness by more than 30–50% while simultaneously reducing deformation and delaying the initiation of fatigue-related damage in highly stressed regions [59].

For rotating components such as drive shafts, a similar effect is achieved through localized wall thickening in critical regions; for example, near flanges or geometric transitions. These reinforcements must be implemented with smooth transitions in order to minimize stress concentrations and reduce the risk of delamination [33,39].

From an application perspective, ribbing represents an effective strategy for increasing the stiffness of CFRP components; however, its efficiency depends strongly on proper integration with the manufacturing process and control of the material microstructure. Inappropriate rib geometry may generate local stress concentrations, non-uniform fibre orientation, manufacturing defects, and increased susceptibility to matrix cracking or delamination, thereby reducing the long-term durability of the component.

### 5.4. Hybrid Metal–Composite Structures

**Figure 18:**
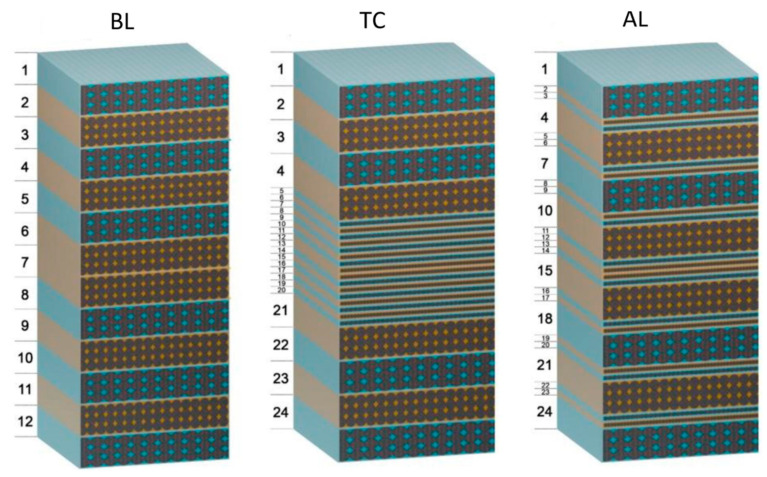
Comparison of different laminate stacking sequences (BL, TC, AL) with varying fibre orientations [7].

**Figure 19:**
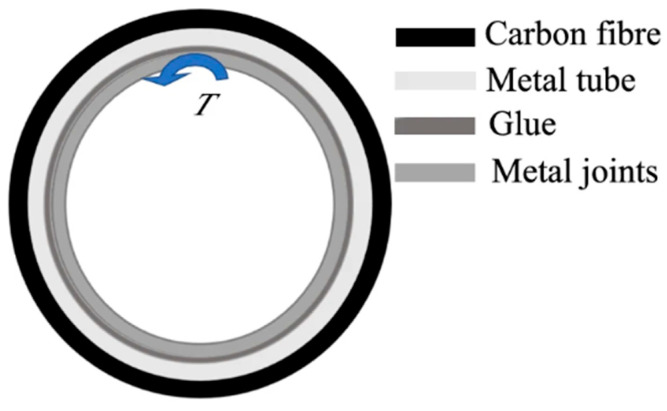
Schematic illustration of a hybrid CFRP–metal structure transmitting torque, including the adhesive joint and metallic elements [5].

Hybrid structures combining CFRP with metallic elements represent (Figure 18) one of the most practical strategies for implementing composite components in automotive powertrain systems. The fundamental principle is to exploit the advantages of both materials—CFRP for low weight and high specific stiffness [25], and metals for regions requiring precise tolerances, high contact resistance, or reliable threaded connections [60,61]. Joints between composites and metals (Figure 19) represent one of the principal limiting factors restricting their wider industrial implementation.

**Figure 20:**
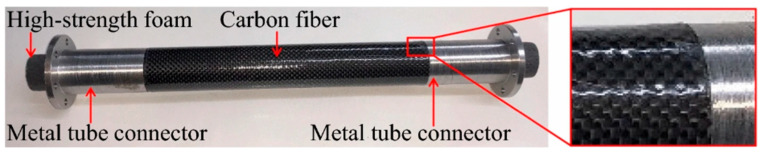
Hybrid CFRP shaft with metallic joints and a foam core, including a detailed view of the material interface [5].

For drive shafts, hybrid structures consisting of CFRP tubes combined with metallic flanges represent a standard engineering solution. The joining process may be realized using adhesive bonding, mechanical fastening, hybrid joints, or overmoulding technologies. From the perspective of fatigue durability, hybrid adhesive–mechanical joints appear to provide the most favourable solution, enabling more uniform stress distribution and improved resistance to failure. Compared with purely mechanical fastening, hybrid adhesive–mechanical joints typically provide more uniform stress transfer and may improve joint fatigue life depending on joint geometry, adhesive properties, surface preparation, and loading conditions [61]. Hybrid structures are implemented in practice, for example, in CFRP drive shafts as illustrated in Figure 20. Additional engineering challenges include galvanic corrosion between carbon fibres and metallic components, surface preparation requirements prior to bonding, and the implementation of non-destructive testing (NDT) methods for quality control of hybrid joints. These factors significantly influence the long-term reliability and industrial applicability of hybrid CFRP–metal structures [60,61].

For gears, a hybrid approach consisting of a steel toothed rim combined with a CFRP web or hub represents an effective solution that combines the high contact resistance of steel with the low weight of composite materials. Such constructions may enable weight reductions of approximately 30–60% compared with fully metallic gear systems while simultaneously reducing vibration transmission and improving NVH performance of the drivetrain [32,36]. For housing and fluid-handling components, the combination of CFRP with metallic inserts enables compliance with requirements related to sealing integrity and dimensional precision. A key design aspect is the minimization of stresses generated by differences in thermal expansion; for example, through the use of transition layers or elastic interlayers. Additional challenges include galvanic corrosion between carbon fibres and metallic components, particularly when aluminium alloys are used, requiring appropriate insulation layers and surface treatment strategies to ensure long-term joint durability [60].

From an application perspective, hybrid structures represent the most realistic approach for implementing CFRP materials in automotive powertrain systems; however, their reliability strongly depends on joint quality, load transfer efficiency, stress management within transition regions, and long-term durability under cyclic thermomechanical loading conditions. Consequently, the development of robust and reproducible joining technologies remains one of the key challenges for their broader industrial adoption. A summary of the most important design strategies used for the optimization of CFRP powertrain components is presented in Table 4.

The comparison of individual design strategies demonstrates that the optimization of CFRP powertrain components cannot rely on a single approach, but instead requires the combination of multiple design principles. Fibre orientation determines the fundamental mechanical properties, thickness gradation enables efficient material distribution, ribbing increases local stiffness, and hybrid structures ensure functional integration with metallic elements. However, the effectiveness of these strategies strongly depends on the specific functional requirements, operating conditions, manufacturing constraints, and dominant degradation mechanisms associated with each component category. As summarized in Table 5 and Table 6, the design of CFRP powertrain components therefore requires an integrated engineering approach combining material selection, reinforcement architecture, manufacturing strategy, and long-term durability considerations. The proposed framework links operating conditions and mechanical requirements with suitable CFRP systems, fibre architectures, manufacturing technologies, and dominant failure mechanisms for representative automotive powertrain applications.

The most effective solutions therefore result from combining these strategies, while their implementation is strongly dependent on manufacturing capabilities and operational requirements. A key limitation remains the integration of design methodologies with real manufacturing constraints, which often determine the practical feasibility of the optimized solution.

## 6. Manufacturing Processes for Functional Composite Powertrain Components

The selection of a manufacturing process is equally as important as material selection for CFRP powertrain components, since it fundamentally influences mechanical properties, void content, fibre orientation, fibre–matrix interface quality, and the final dimensional accuracy of the component. Manufacturing technology also determines process economics, production cycle time, and the possibility of integrating complex geometrical features into a single part.

For the design of CFRP powertrain components, it is therefore necessary to consider not only the required mechanical performance but also the technological limitations of the selected manufacturing process. Individual technologies differ significantly in terms of suitability for short or continuous fibres, achievable void content, laminate consolidation quality, and large-scale production capability.

The selection of a manufacturing route is further influenced by the functional category of the component, since highly loaded rotating and structural components generally require continuous-fibre processing technologies with superior laminate quality, whereas housing and fluid-handling components can often be manufactured using short- or long-fibre thermoplastic processes optimized for high-volume production.

This section summarizes the principal manufacturing technologies relevant to functional CFRP applications in automotive powertrain systems, focusing on their technological principles, manufacturing capabilities, limitations, production economics, and suitability for specific material systems and component categories.

### 6.1. Injection Moulding

Injection moulding represents the most widely used manufacturing process for thermoplastic CFRP composites reinforced with short or long fibres. The process consists of pellet plasticization followed by injection into a mould cavity under high pressure, where the component cools and solidifies. Owing to short cycle times on the order of tens of seconds, this method is highly suitable for high-volume automotive production [48]. A major advantage of the process is its high degree of automation and excellent production repeatability [67].

The principal advantage of injection moulding lies in its ability to produce geometrically complex components in a single step, including integrated channels, ribs, threaded inserts, and mounting features. Consequently, this process is widely applied for housings, manifolds, oil pans, and other components with high geometrical complexity [67]. Compared with conventional metallic components, injection-moulded CFRP parts may provide weight reductions of approximately 20–50% depending on component geometry and material system, while simultaneously reducing the number of assembly operations through functional integration [67].

A critical aspect of the process is fibre orientation during mould filling, which leads to anisotropic mechanical properties. Fibres tend to align predominantly along the melt flow direction near the walls and perpendicular to it in the core region, producing a characteristic layered structure with non-uniform property distribution. Accurate prediction of mechanical behaviour therefore requires melt flow simulation coupled with structural analysis of the component [67].

The main limitation of injection-moulded CFRP composites is the reduced effective fibre length and lower mechanical performance compared with continuous-fibre laminates. Fibre breakage occurring during processing decreases the efficiency of load transfer. Another significant challenge is the control of deformation caused by shrinkage and material anisotropy.

A variant of this process is overmoulding, which enables the combination of thermoplastic matrices with metallic or continuous-fibre preforms in a single manufacturing step, resulting in hybrid structures that combine the geometric flexibility of injection moulding with the superior stiffness and strength of continuous-fibre reinforcements [48,68].

Representative material systems and typical processing characteristics for injection-moulded CFRP composites used in automotive powertrain applications are summarized in Table 5.

### 6.2. Compression Moulding and Compression Forming

Compression moulding represents the preferred manufacturing method for continuous-fibre CFRP composites requiring high mechanical performance. The process involves consolidation of prepregs or woven fabrics within a mould under the combined action of temperature and pressure, enabling low void content and high laminate quality [69]. Owing to the excellent fibre–matrix interface quality and precise control of laminate stacking, this technology is among the most important methods for manufacturing highly loaded structural components [69].

For thermoset systems, curing is performed at elevated temperatures, whereas thermoplastic systems such as PEEK and PPS employ hot compression moulding followed by controlled cooling [70]. Cooling rate significantly affects matrix crystallinity and consequently the resulting mechanical properties of the composite [37]. Higher crystallinity improves stiffness and thermal stability but may reduce material toughness.

Typical crystallinity levels achieved in CF/PEEK and CF/PPS laminates range approximately between 25 and 40%, depending on processing temperature, consolidation conditions, and cooling rate. Appropriate control of crystallinity is therefore essential for achieving the desired balance between stiffness, toughness, creep resistance, and thermal stability [37].

Compression moulding is primarily employed for components requiring high strength and fatigue durability, such as drive shafts, gears, and structural powertrain components. A major advantage of the process is the possibility of precise fibre orientation control and optimization of laminate architecture according to specific loading conditions. Compared with injection moulding, compression moulding generally provides substantially lower void content, improved fibre alignment, and superior mechanical performance; however, these advantages are achieved at the expense of longer cycle times and reduced suitability for high-volume production [69].

The disadvantages of compression moulding include longer production cycles, higher energy consumption, and lower suitability for mass production compared with injection moulding [69]. The process also requires precise control of temperature, pressure, and consolidation conditions, since insufficient processing may result in elevated void content, incomplete consolidation, interlaminar delamination, and reduced long-term fatigue durability of the component.

### 6.3. Overmoulding and Hybrid Manufacturing Processes

Overmoulding represents a hybrid manufacturing approach combining continuous-fibre preforms with injection moulding of a thermoplastic matrix. The process involves placing a preform into the mould cavity followed by over-injection of molten polymer, which fills geometrically complex regions and ensures adhesion between different sections of the component [68].

This approach combines the high mechanical performance of continuous fibres with the manufacturing flexibility of injection moulding. Compared with purely injection-moulded short-fibre composites, overmoulded structures can achieve significantly higher local stiffness and load-carrying capacity while retaining the ability to manufacture geometrically complex functional features within a single component [68].

A significant advantage of overmoulding is the possibility of integrating metallic inserts, mounting elements, and functional details directly during manufacturing. This reduces the number of assembly operations and improves overall component integration. Hybrid manufacturing approaches also enable local optimization of material properties according to specific loading conditions.

A key factor is the compatibility between preform and matrix materials, which fundamentally influences bonding quality and the resulting mechanical properties of the component [70]. Insufficient adhesion may lead to interlaminar failure, progressive debonding, reduced fatigue durability, or premature separation between individual sections of the component during long-term service operation.

The quality of the preform–matrix interface directly determines load transfer efficiency between continuous-fibre and overmoulded regions. Poor interfacial bonding may lead to stress concentrations, localized debonding, and premature failure under cyclic mechanical loading conditions [70].

### 6.4. Additive Manufacturing

Additive manufacturing of CFRP composites, particularly FDM-based technologies reinforced with short or continuous fibres, has emerged as a flexible manufacturing route for prototyping, tooling, and small-series production of geometrically complex components. The principal advantages include high geometrical flexibility, rapid fabrication of functional prototypes, and minimal material waste [4].

Components reinforced with continuous fibres exhibit significantly improved mechanical properties compared with unreinforced polymers; however, they still remain inferior to laminates manufactured using conventional techniques due to higher void content and less homogeneous microstructure. Continuous-fibre additively manufactured composites may achieve tensile strengths exceeding 600–800 MPa depending on fibre content and printing strategy; however, these values generally remain below those of conventionally consolidated laminates due to higher porosity and less efficient fibre packing [39]. A major limitation remains the relatively weak interlayer bonding strength, which promotes anisotropic behaviour and may accelerate delamination and fatigue damage under cyclic loading conditions.

For high-temperature materials such as PEEK and PEKK, the process becomes technologically demanding because of elevated processing temperatures and strict requirements for thermal field control during printing. Insufficient process control may result in deformation, residual stresses, or inadequate layer consolidation.

At present, additive manufacturing is primarily utilized for prototyping, tooling production, and low-volume functional applications, whereas its implementation in large-scale manufacturing of highly loaded powertrain components remains limited by production rate, interlayer bonding quality, and process consistency [39]. Future industrial adoption will depend on further improvements in continuous-fibre deposition technologies, process monitoring, thermal management, and the processing of high-performance thermoplastics such as PEEK, PEKK, and PPS. A comparison of the principal manufacturing technologies used for CFRP powertrain components is presented in Table 7.

The comparison of manufacturing processes demonstrates that technology selection has a fundamental influence on the resulting mechanical properties, laminate quality, fibre orientation, void content, and production economics of CFRP powertrain components. Injection moulding is particularly suitable for geometrically complex, high-volume components manufactured from short- or long-fibre thermoplastic composites, whereas compression moulding provides superior laminate quality and mechanical performance for continuous-fibre structural applications. Overmoulding represents an intermediate solution that combines the manufacturing flexibility of injection moulding with the high stiffness and strength of continuous-fibre reinforcements, enabling efficient production of hybrid structures. Additive manufacturing currently remains primarily focused on prototyping, tooling production, and low-volume functional applications; however, ongoing developments in continuous-fibre deposition, process monitoring, and high-temperature thermoplastic processing may further expand its applicability for advanced automotive powertrain components. Consequently, manufacturing process selection represents a critical engineering decision that must simultaneously consider performance requirements, production volume, component complexity, and economic constraints.

As summarized in Table 8, the selection of manufacturing technology is closely linked with fibre architecture, component functionality, and dominant degradation mechanisms. Different processing routes provide substantially different levels of laminate quality, fibre orientation control, void content, and long-term structural reliability, which directly influence the durability and operational performance of CFRP powertrain components.

## 7. Failure Mechanisms and Durability Under Powertrain Conditions

Understanding the failure mechanisms of CFRP powertrain components is essential for reliable design and service life prediction of composite systems. During operation, powertrain components are exposed to combined mechanical, thermal, chemical, and tribological loading, with individual degradation processes often occurring simultaneously and interacting with one another. Unlike metallic materials, where failure is frequently governed by a single dominant mechanism, composite materials exhibit multi-stage damage involving the simultaneous degradation of several microstructural constituents.

Damage in CFRP composites typically begins at the microscale through the initiation of cracks in the polymer matrix or weakening of the fibre–matrix interface. This is followed by progressive damage propagation in the form of interlaminar delamination, fibre debonding, and localized fracture of the carbon fibres themselves. The nature and rate of degradation strongly depend on matrix type, fibre orientation, laminate architecture, and component operating conditions. Consequently, failure behaviour cannot be attributed solely to material selection but must be evaluated as the combined result of material system characteristics, reinforcement architecture, manufacturing quality, and operational loading conditions.

The anisotropic nature of CFRP materials is also a significant factor. Mechanical properties strongly depend on the loading direction relative to fibre orientation, meaning that a component may exhibit high strength in one direction while simultaneously showing lower resistance to interlaminar shear or transverse loading. Critical regions typically include joints, holes, geometric transitions, and stress concentration areas, where local load redistribution and damage initiation occur.

Under powertrain operating conditions, the combined effects of temperature and operating fluids also play an important role. Elevated temperatures may lead to matrix plasticization, reduction of the glass transition temperature, and decreased interfacial adhesion. At the same time, absorption of oils, fuels, or coolants may accelerate chemical degradation of the polymer matrix and promote microcrack formation. Under cyclic mechanical loading, these damage mechanisms progressively accumulate, leading to stiffness degradation and reduced component service life.

This section summarizes the principal failure mechanisms relevant to automotive powertrain operating conditions, emphasizing the relationships between damage initiation, propagation mechanisms, service loading conditions, and their influence on the long-term durability and reliability of CFRP components.

### 7.1. Creep Failure

Creep failure of CFRP composites in powertrain systems arises from the combined effects of long-term mechanical loading and elevated temperature during service. At the microstructural level, creep is governed by viscoelastic deformation of the amorphous phase of the polymer matrix and viscoplastic processes associated with the reorganization of crystalline regions in semi-crystalline materials such as PEEK and PPS [25]. The quality of the fibre–matrix interface and the ability of the matrix to transfer shear stresses between individual laminate layers also play a significant role [70].

For CF/PEEK composites, it has been demonstrated that temperatures close to Tg lead to a gradual decrease in torsional stiffness over time. For example, at 140 °C and under a load corresponding to 30% of the short-term strength, the effective elastic modulus decreases by approximately 20–35% after 1000 h. Such stiffness degradation may directly influence dimensional stability, shaft torsional rigidity, sealing integrity, and the long-term operational reliability of powertrain components [48].

This behaviour is associated with increased mobility of polymer chains, which enables time-dependent deformation processes. Under long-term loading, creep may result in loss of preload in bolted joints, deformation of flanges, and reduced sealing performance of components.

A particularly critical regime is the interaction between creep and fatigue, referred to as the creep–fatigue mechanism. For double Cardan CFRP drive shafts operating under combined torsional cyclic loading and elevated temperature conditions, creep–fatigue interaction has been reported to accelerate damage accumulation, potentially reducing component lifetime by approximately 40–60% compared with fatigue loading alone [34]. This effect is caused by accelerated propagation of interlaminar cracks due to creep deformation of the matrix during cyclic loading. The most critical regions are stress concentration areas such as joints, mounting holes, and geometric transitions.

To minimize creep damage, components must be designed so that the majority of the load is carried by the fibres rather than the matrix. From a materials perspective, the use of semi-crystalline matrices with higher Tg values is advantageous, together with maintaining a sufficient temperature margin between service temperature and material Tg [70]. Fibre orientation optimization, thickness tailoring, and appropriate laminate architecture design play an important role in minimizing interlaminar shear stresses and delaying creep-induced damage accumulation, demonstrating the strong relationship between design strategy and long-term durability.

### 7.2. Fatigue Cracking

Fatigue damage in CFRP composites occurs through the gradual accumulation of microdamage in several successive stages. In the initial stage, matrix microcracks form, particularly in plies oriented transverse to the principal loading direction (90°), gradually leading to the characteristic damage state (CDS) [58]. These microcracks do not initially cause immediate component failure, but they create favourable conditions for further damage progression [64]. Although the laminate may continue to carry load after reaching the characteristic damage state, the presence of matrix microcracks facilitates stress redistribution and accelerates the initiation of interlaminar damage mechanisms during subsequent fatigue loading.

In the subsequent stage, damage propagates through fibre–matrix interfacial debonding and the growth of interlaminar delaminations, leading to progressive reduction in laminate stiffness. A decrease in elastic modulus of approximately 10–15% is often considered a limiting condition prior to component failure [64]. Such stiffness degradation may adversely affect vibration behaviour, dimensional stability, and load transfer capability in highly loaded drivetrain components before final structural failure occurs. In the final stage, rapid damage growth occurs, accompanied by loss of load redistribution capability between fibres and final fibre fracture.

Temperature has a significant influence on fatigue life. An increase in temperature of 10 °C may reduce fatigue life by 15–25%, which is associated with reduced matrix stiffness and accelerated degradation processes at the fibre–matrix interface [33]. The absorption of operating fluids also has a negative effect, as it may cause matrix plasticization and accelerate microcrack initiation.

Fibre orientation and laminate stacking sequence are also important factors. Laminates with a high proportion of 0° plies exhibit high fatigue strength in the loading direction but lower resistance to interlaminar failure. In contrast, multidirectional laminates provide improved stress redistribution but may be more susceptible to delamination.

Fatigue resistance can be improved through matrix modification; for example, by incorporating graphene nanoplatelets or oxidized nanostructures, which increase resistance to crack propagation and slow damage progression [64]. The use of toughening agents, nanoparticle modification, and optimization of the fibre–matrix interface can significantly delay crack propagation and damage accumulation, demonstrating that fatigue durability depends not only on material selection but also on laminate design and microstructural engineering.

### 7.3. Delamination Failure

Delamination is one of the most critical failure mechanisms in laminated CFRP composites. It involves the separation of individual laminate layers caused by interlaminar shear or normal stresses. Delamination failure is particularly dangerous because it can substantially reduce the residual strength of a component without visible surface damage [64]. In highly loaded drivetrain components, delamination may result in a progressive reduction of stiffness, altered vibration behaviour, loss of load transfer capability between plies, and accelerated fatigue damage accumulation prior to final structural failure.

Delamination is most commonly initiated in stress concentration regions such as holes, laminate edges, geometric transitions, or areas with abrupt changes in fibre orientation. The presence of manufacturing defects, such as voids or locally insufficiently consolidated layers, also plays an important role. Under dynamic or impact loading, barely visible impact damage (BVID) may occur, where the component surface remains relatively intact while extensive interlaminar damage develops inside the laminate [65].

Delamination propagation is characterized by fracture toughness under different failure modes: Mode I (opening), Mode II (sliding shear), and mixed-mode loading. For powertrain components, combined loading modes are particularly critical, since bending, torsion, and vibration may act simultaneously [65].

**Figure 21:**
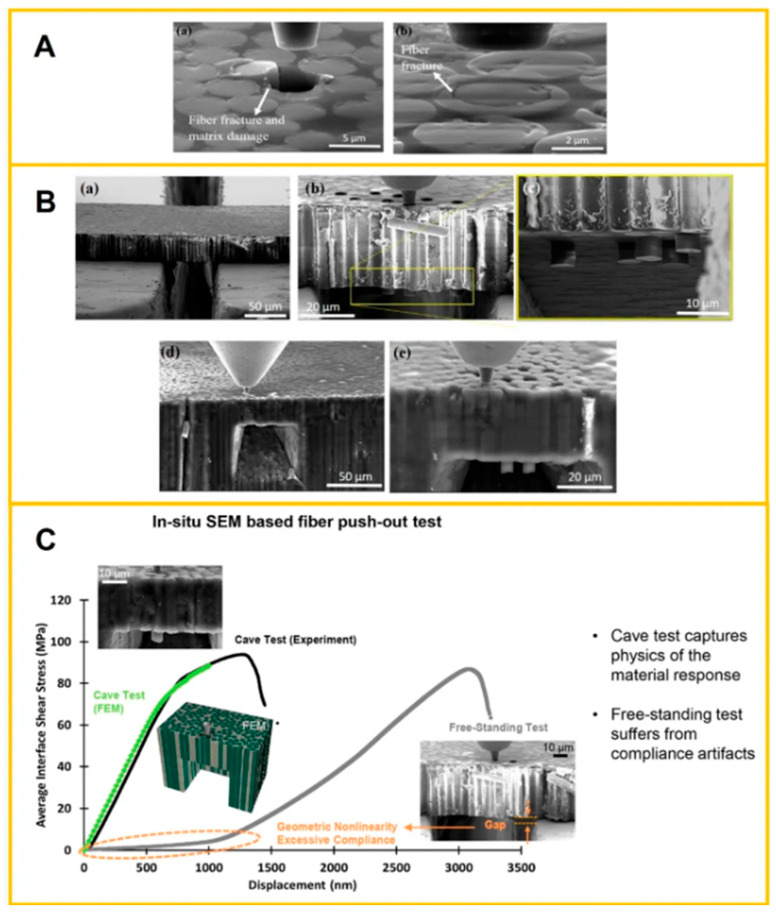
Mechanical behaviour of the fibre–matrix interface analysed using SEM observations and the fibre push-out test. (**A**) Local damage mechanisms observed during the fibre push-out test: (**a**) SEM image showing fibre fracture accompanied by local matrix damage after fibre push-out, and (**b**) higher-magnification view of fibre fracture. (**B**) In situ SEM observations of the fibre push-out test: (**a**) preparation of the free-standing specimen with a machined cavity, (**b**) loading configuration during fibre push-out with the indenter positioned above the selected fibre, (**c**) enlarged view of the fibre array and supporting cavity prior to loading, (**d**) progressive fibre displacement and interfacial debonding during loading, and (**e**) final stage of the fibre push-out test after complete fibre displacement. (**C**) Comparison of the in situ SEM-based cave test and the conventional free-standing fibre push-out test: experimentally measured and FEM-predicted average interfacial shear stress–displacement responses together with representative SEM images illustrating the different deformation mechanisms and the influence of specimen compliance [41].

Delamination resistance can be increased through laminate stacking optimization, the use of toughened matrices, or local reinforcement of critical regions. Effective solutions also include thin-ply laminate architectures or interleaf layers, which reduce interlaminar stress concentrations and slow crack growth [66]. Nanoparticle modification of the matrix also offers significant potential by increasing interlaminar fracture toughness and improving the stability of the fibre–matrix interface [41]. Figure 21 illustrates the mechanical behaviour of the fibre–matrix interface analyzed using SEM observations and fibre push-out testing, highlighting the interfacial damage mechanisms that govern crack initiation, interlaminar failure, and subsequent delamination propagation in CFRP laminates.

The failure mechanisms of CFRP composites in automotive powertrain systems result from a complex interaction of mechanical, thermal, chemical, and tribological loading. Under real operating conditions, a single degradation mechanism rarely acts independently; instead, multiple mechanisms interact simultaneously and contribute to cumulative damage development. Creep deformation may accelerate delamination, fatigue damage reduces interlaminar strength, and chemical degradation of the matrix promotes the initiation of microcracks.

The long-term reliability of CFRP components therefore depends not only on the intrinsic material properties but also on optimization of laminate architecture, manufacturing quality, joint design, and the ability to predict damage progression throughout the service life of the component. Consequently, durability must be considered as the combined outcome of material selection, reinforcement architecture, manufacturing quality, and component design strategy rather than as an inherent material characteristic alone. A major research challenge remains the development and experimental validation of predictive numerical models capable of reliably simulating coupled thermomechanical, chemical, tribological, and fatigue-related degradation processes under realistic automotive powertrain operating conditions.

### 7.4. Wear Under Powertrain Operating Conditions

Wear of CFRP composites in powertrain systems represents a complex tribological process dependent on the combination of loading, sliding speed, temperature, and lubrication conditions. Three dominant mechanisms are generally observed: adhesive wear, abrasive wear, and surface contact fatigue [59]. The severity of wear strongly depends on contact pressure, lubrication regime, fibre orientation, matrix type, and operating temperature. Consequently, tribological performance cannot be evaluated independently of the overall component design and service environment.

Adhesive wear occurs during direct contact with a metallic counterface, where local material transfer and the formation of a so-called transfer film may occur, potentially stabilizing tribological behaviour [9]. Abrasive wear is caused by detached fibre and matrix fragments acting as third-body particles, with this mechanism being particularly critical in contact with soft metals such as aluminium alloys. In severe cases, exposed carbon fibres may act as hard abrasive particles that accelerate counter-surface wear and progressively increase friction instability within the tribological system.

Under cyclic contact loading, pitting failure may also develop, initiated by subsurface cracks in the fibre–matrix interface region. This mechanism is typical for gears and components exposed to repeated contact loading [65]. Wear resistance can be improved through optimization of fibre orientation, incorporation of solid lubricants, surface modification techniques, and appropriate selection of matrix systems with enhanced tribological stability under elevated temperatures. A summary of the principal degradation mechanisms of CFRP composites relevant to powertrain operating conditions, including critical damage regions and possible mitigation strategies, is presented in Table 9.

## 8. Future Trends

The future implementation of CFRP composites in automotive powertrain systems will be determined by the combination of material innovations, advanced manufacturing technologies, and the transformation of modern vehicle architectures. Current developments indicate that the next generation of CFRP components will not be optimized solely for weight reduction, but will instead be designed as multifunctional systems integrating mechanical, thermal, vibrational, and environmental requirements throughout the entire component life cycle.

One of the main factors influencing future development is the transition toward electrification. Electric powertrain systems operate at lower maximum temperatures than internal combustion engines, thereby expanding the application potential of CFRP composites even for materials with lower thermal resistance. At the same time, electric propulsion systems introduce new requirements related to high-speed rotating systems, dynamic loading, and vibroacoustic behaviour (NVH). In this area, CFRP composites provide significant advantages owing to their high stiffness-to-weight ratio and superior vibration damping compared with metallic materials [66,71]. At the same time, battery systems, power electronics housings, and electric drive units introduce new requirements related to electromagnetic compatibility, thermal management, and dimensional stability under cyclic thermal loading, creating additional opportunities for multifunctional CFRP-based solutions.

Another important trend is the transition from monolithic composite structures toward hybrid multi-material systems combining CFRP with metallic elements. Such solutions enable efficient functional distribution between materials—CFRP provides low weight and high specific stiffness, while metallic regions ensure high contact strength and wear resistance. This approach appears particularly promising for rotating components, gear systems, and hybrid drive shafts, where fully CFRP-based solutions still face limitations associated with contact strength and tribological stability [62,63].

Digitalization of composite structure design and the application of advanced numerical tools are also becoming increasingly important. The combination of finite element analysis, topology optimization, and data-driven approaches enables optimization of fibre orientation, stacking sequence, and local laminate thickness for complex multiaxial loading conditions while minimizing component weight [72,73]. In the future, increasing implementation of the digital twin concept can be expected, enabling continuous prediction of damage evolution, remaining service life, and optimization of CFRP component operation. The integration of digital twins with structural health monitoring systems may further enable predictive maintenance strategies and condition-based service scheduling for future composite powertrain components.

From a materials perspective, a clear shift toward recyclable and sustainable composite systems is evident, particularly high-performance thermoplastics and vitrimer materials. Systems such as CF/PEEK, CF/PPS, and CF/PEKK allow repeated processing without significant degradation of properties, while vitrimer composites additionally provide the possibility of repair and chemical recycling [30,31]. Despite substantial progress, the insufficiently investigated long-term behaviour of these systems under combined thermal, mechanical, and chemical loading remains a major challenge.

A key factor for broader industrial implementation of CFRP composites is the optimization of manufacturing processes and reduction of production costs. Technologies such as automated fibre placement (AFP), high-speed thermoplastic compression moulding, and hybrid overmoulding processes enable improvements in laminate quality, production reproducibility, and reduction of void content [58,61]. At the same time, additive manufacturing of continuous-fibre composites is gaining importance and may enable production of geometrically complex components with locally optimized reinforcement architectures in the future.

For improved comparison of individual CFRP systems in terms of thermal, mechanical, and operational performance, Table 10 presents an integrated overview of the most significant material solutions applicable to automotive powertrain systems.

The final properties of CFRP components are also strongly influenced by the selected manufacturing process, which affects laminate consolidation quality, void content, and fibre orientation. A comparison of the principal manufacturing technologies used for CFRP powertrain components is presented in Table 11.

Overall, the future development of CFRP composites in powertrain systems is expected to move toward intelligent, multifunctional, and sustainable material systems integrated into digitally controlled design and manufacturing processes. The greatest industrial potential is expected in hybrid multi-material architectures, recyclable thermoplastic and vitrimer systems, advanced automated manufacturing technologies, and digitally optimized composite structures capable of simultaneously satisfying mechanical, economic, and sustainability requirements of next-generation electrified powertrain systems.

The identified trends indicate that the future development of CFRP composites for automotive powertrain systems will be shaped primarily by the combination of novel material solutions, advanced manufacturing technologies, and the digitalization of the design process. Electrification is changing the nature of operating conditions, while new high-temperature polymers and multifunctional composites are expanding the application potential of CFRP materials into more demanding environments. The successful implementation of these emerging material systems will depend not only on their intrinsic properties but also on the availability of scalable manufacturing technologies capable of ensuring consistent laminate quality, low void content, and economically viable production.

At the same time, the increasing emphasis on recyclability and the circular economy is shifting attention toward thermoplastic and vitrimer systems, which enable more efficient material life cycles. Digitalization and the application of artificial intelligence also play a key role in optimizing component design, manufacturing processes, and service life prediction.

Overall, future CFRP powertrain components are expected to evolve into multifunctional, digitally optimized, and sustainable engineering systems that integrate structural performance, durability, manufacturability, recyclability, and life-cycle considerations within a unified design framework.

## 9. Discussion

The analysis of CFRP composite materials for automotive powertrain applications demonstrates that current developments in automotive composites are strongly driven by increasing demands for vehicle lightweighting, improved energy efficiency, and emission reduction. Othman et al. [1] report that CFRP materials represent one of the most promising alternatives to conventional steel and aluminium components, particularly in applications requiring a high strength-to-weight ratio. A similar perspective is presented by Khatib et al. [2], who emphasize that reducing the weight of the powertrain system not only decreases energy consumption but also reduces inertial forces and improves the dynamic behaviour of the vehicle. The importance of lightweight composite structures is further increasing in the context of electromobility, where weight reduction directly affects vehicle driving range, energy efficiency, and the vibroacoustic behaviour of electric propulsion systems [20]. Czerwinski [19] additionally points out that future automotive lightweighting strategies will increasingly depend on the ability to combine weight reduction with manufacturing efficiency and environmental sustainability, which significantly favours advanced composite materials.

Despite these significant advantages, the analysed studies indicate that the implementation of CFRP composites into powertrain systems represents a substantially more complex challenge than their application in conventional load-bearing vehicle structures. Hamzat et al. [4] emphasize that degradation of CFRP composites under real operating conditions occurs through the interaction of multiple coupled mechanisms involving fatigue damage, creep processes, thermal cycling, oxidation, and tribological wear. This represents a fundamental difference compared with traditional metallic materials, where failure is often governed by a single dominant mechanism. Wazeer et al. [20] report that in CFRP systems simultaneous degradation of the matrix, fibre–matrix interface, and fibres themselves occurs, while the individual degradation mechanisms mutually accelerate one another. Consequently, the suitability of a CFRP system cannot be evaluated solely based on its maximum mechanical properties, but rather on the stability of its behaviour under long-term combined loading conditions.

Conventional CF/epoxy systems, despite the increasing importance of thermoplastic composites, still maintain a dominant position in many applications due to their well-established manufacturing technologies, low void content, and favourable cost-to-performance ratio. Alshammari et al. [10], however, point out that epoxy matrices exhibit limited thermal stability and sensitivity to degradation during long-term exposure to elevated temperatures and operating fluids. Guo et al. [40] demonstrated that combined thermal cycling and chemical degradation lead to weakening of the fibre–matrix interface, reduction of Tg, and accelerated initiation of delamination. Similar conclusions were presented by Basha et al. [41], who reported significant reductions in the mechanical stability of CFRP laminates after prolonged exposure to elevated temperatures. These findings suggest that although conventional epoxy systems provide excellent static mechanical properties, their long-term stability under aggressive powertrain operating conditions remains a limiting factor for broader application.

For these reasons, the importance of high-performance thermoplastic matrices, particularly PEEK, PPS, and PEKK, has significantly increased in recent years. Yao et al. [11] and Wang et al. [23] state that thermoplastic CFRP composites combine high thermal and chemical resistance with improved toughness and impact resistance compared with thermoset systems. Dai et al. [32] experimentally demonstrated that CF/PEEK composites maintain high strength, tribological stability, and wear resistance even at temperatures where epoxy matrices already undergo severe degradation. Gabrion et al. [9] further emphasize that high-temperature thermoplastic composites exhibit more favourable thermomechanical behaviour under prolonged thermal loading, which is particularly critical for rotating and tribologically stressed powertrain components.

A significant disadvantage of CF/PEEK systems, however, remains their high cost and technological processing complexity. Wang et al. [23] note that processing temperatures exceeding 360 °C substantially complicate laminate consolidation, increase manufacturing energy demand, and limit the economic feasibility of large-scale production. From a practical perspective, CF/PPS systems therefore represent a considerably more attractive compromise between thermal stability, mechanical performance, and manufacturing efficiency. Morgado et al. [45] demonstrated very good fatigue resistance of CF/PPS composites under cyclic loading, while Chen et al. [46] highlighted their excellent chemical stability and low absorption of operating fluids. Wan et al. and Takahashi et al. [28] even identified PPS systems as among the most promising materials for large-scale automotive CFRP production due to their more favourable processing window and lower manufacturing costs. The literature analysis therefore indicates that future development will not converge toward a single universally dominant material, but rather toward application-specific material solutions optimized for individual operating zones of the powertrain system.

An important observation emerging from the analysed studies is that the service life of CFRP components is determined to a much greater extent by microstructural degradation mechanisms than by the nominal mechanical properties of the material itself. Hussain et al. [8] report that fatigue damage in CFRP composites develops through gradual accumulation of microdamage in the form of matrix cracking, fibre–matrix debonding, and subsequent laminate delamination. Gao et al. [69] emphasize that damage progression strongly depends on laminate architecture, interface quality, and operating conditions. Guo et al. [68] further point out that creep-fatigue loading results in substantially faster damage growth compared with the isolated action of individual mechanisms. This effect is particularly critical for components exposed to elevated temperatures and long-term cyclic loading, such as drive shafts and transmission systems. Consequently, conventional laboratory testing performed at room temperature cannot adequately predict the behaviour of CFRP components under real powertrain operating conditions.

The quality of the fibre–matrix interface plays a crucial role, representing a critical region for damage initiation. De Leon et al. [5] emphasize that interfacial adhesion directly affects load transfer efficiency and resistance to delamination. Liu et al. [71] report that modern approaches based on plasma activation, graphene nanoplatelets, or carbon nanotubes significantly improve interfacial adhesion while simultaneously slowing damage progression under cyclic loading. Rahman et al. [43] additionally demonstrated that appropriately designed vitrimer interfaces can significantly enhance crack propagation resistance and improve recyclability of the composite system. These findings indicate that future development of CFRP composites will not rely solely on optimization of the matrix or fibres themselves, but primarily on engineering of the fibre–matrix interface as a decisive factor governing long-term reliability.

An additional factor significantly influencing the mechanical behaviour of CFRP components is the design of laminate architecture and fibre orientation. Xu et al. [33] demonstrated that stacking sequence optimization strongly affects torsional stiffness, fatigue life, and dynamic stability of rotating components. Similar conclusions were reported by Maccioni et al. [49] and Černe et al. [58], who demonstrated a significant influence of fibre orientation on contact strength and delamination progression in CFRP gears. The literature analysis indicates that the mechanical behaviour of CFRP components is determined not only by intrinsic material properties, but primarily by the ability to optimize local fibre distribution according to dominant stress fields. Consequently, numerical optimization tools, topology optimization, and data-driven design approaches are becoming increasingly important.

Manufacturing technology itself also plays a crucial role. Wu et al. [24] point out that manufacturing defects, particularly voids, insufficient consolidation, or local fibre misalignment, have a substantial influence on the fatigue life of CFRP composites. Wang et al. [23] emphasize that injection moulding enables efficient large-scale production of complex components, albeit at the cost of reduced mechanical performance caused by shorter effective fibre lengths. In contrast, compression moulding of continuous-fibre prepregs provides significantly higher mechanical performance, although with higher production costs and lower productivity. Hybrid manufacturing technologies and overmoulding therefore represent promising future directions, combining the high mechanical performance of continuous fibres with the flexibility of thermoplastic processing technologies [23,24].

A significant shift can also be observed in the area of environmental sustainability and circular economy. Aldosari et al. [3] and Tran et al. [12] point out that conventional thermoset composites represent a major challenge from the recycling perspective because the crosslinked matrix structure practically prevents material reprocessing. Kang et al. [22] emphasize the growing potential of vitrimer composites, which combine the mechanical performance of thermosets with the possibility of repair, reshaping, and chemical recycling. Similarly, Mandal et al. [15] identify vitrimer systems and self-healing mechanisms as one of the most promising future directions in CFRP structure development. This trend suggests that future CFRP systems will not be optimized solely in terms of mechanical performance, but also with regard to material life cycle and environmental sustainability.

Beyond recyclability, increasing importance can also be expected for intelligent and multifunctional composites. Wang et al. [23] state that future CFRP components will increasingly integrate sensing and monitoring functionalities enabling real-time tracking of deformation, damage initiation, and local temperature changes. The combination of composite structures with digital twins, numerical optimization, and artificial intelligence algorithms may fundamentally transform the design and maintenance of automotive powertrain systems. In this context, CFRP components will no longer represent merely passive structural elements, but rather intelligent multifunctional systems capable of continuous self-diagnostics and prediction of remaining service life. The overall analysis indicates that the successful implementation of CFRP composites in automotive powertrain systems requires simultaneous optimization of four interrelated domains: operational requirements, material selection, manufacturing technology, and durability performance. High-temperature thermoplastic systems such as CF/PEEK and CF/PPS provide superior thermal and chemical stability, whereas manufacturing technologies such as compression moulding and overmoulding enable the laminate quality required for highly loaded applications. At the same time, long-term reliability remains governed by fatigue damage, delamination, creep behaviour, and tribological degradation, emphasizing the necessity of an integrated engineering approach throughout the entire component life cycle.

Despite substantial technological progress, implementation of CFRP composites in powertrain systems remains limited by several major challenges. The most significant include high manufacturing costs, technological processing complexity, limited predictability of long-term durability, and insufficient experimental data under combined operating conditions. Most currently available studies are still conducted under simplified laboratory conditions that cannot fully reproduce the complex loading scenarios of real automotive systems. Future research must therefore focus primarily on multiphysical evaluation of CFRP components under combined thermal, chemical, tribological, and dynamic loading, as well as on the development of reliable numerical models capable of accurate long-term life prediction. The ability to integrate material engineering, digital design, advanced manufacturing technologies, and environmental sustainability will ultimately determine the extent of future CFRP implementation in next-generation automotive powertrain systems.

## 10. Conclusions

Carbon fibre-reinforced polymer (CFRP) composites represent a promising class of lightweight structural materials for automotive powertrain systems, where increasing demands for weight reduction, energy efficiency, and emission reduction are accelerating the transition from conventional metallic materials toward advanced composite solutions. However, the successful implementation of CFRP components is strongly constrained by the complex operating environment of powertrain systems, involving elevated temperatures, chemical exposure, cyclic loading, creep deformation, and tribological stresses. These factors require a comprehensive optimization of material selection, reinforcement architecture, manufacturing technology, and durability performance.

The analysis presented in this review demonstrates that no universal CFRP material system exists for all powertrain applications. High-performance thermoplastic composites, particularly CF/PEEK, CF/PPS, and CF/PEKK, offer significant advantages in terms of thermal stability, chemical resistance, and long-term durability, while conventional CF/epoxy systems remain attractive for less demanding applications due to their lower cost and established manufacturing infrastructure. Among the analysed materials, CF/PEEK represents the benchmark solution for high-temperature applications, whereas CF/PPS provides one of the most favourable compromises between performance, processability, and economic efficiency.

The long-term reliability of CFRP components is governed primarily by progressive degradation mechanisms including fatigue damage, creep deformation, delamination, and tribological wear. These mechanisms rarely act independently under real operating conditions and are strongly influenced by fibre orientation, laminate architecture, manufacturing quality, and the integrity of the fibre–matrix interface. Consequently, durability must be considered as the combined outcome of material selection, structural design, manufacturing process, and service environment.

The review further demonstrates that reinforcement architecture, laminate optimization, hybrid metal–composite structures, and advanced manufacturing technologies play a decisive role in achieving the required balance between weight reduction, mechanical performance, durability, and production feasibility. In particular, hybrid structures combining CFRP and metallic elements currently represent one of the most realistic approaches for industrial implementation in automotive powertrain systems.

Future developments are expected to focus on recyclable thermoplastic and vitrimer systems, advanced automated manufacturing technologies, intelligent multifunctional composites, and digitally assisted design approaches. The integration of material engineering, manufacturing optimization, durability prediction, and sustainability considerations will be essential for the broader adoption of CFRP composites in next-generation electrified powertrain systems.

## Figures and Tables

**Table 1:** Representative design requirements and target performance criteria for CFRP composites intended for automotive powertrain applications, synthesized from published studies on thermomechanical behaviour, fatigue performance, tribological properties, and high-performance composite materials [9,30,31,34,38].

Requirement	Typical Value for Powertrain Applications	Note
**Operating temperature**	130–300 °C	Depends on the temperature zone
**Matrix Tg (minimum)**	Toper. +30–50 °C	Safety margin
**Oil absorption**	<1 wt.%/1000 h	At operating temperature
**Fatigue (HCF)**	>10^7^ cycles at 80% σmax	For drive shafts
**Creep**	<0.5%/1000 h at 30% σult	At maximum temperature
**Tan δ**	0.01–0.05	Depends on fibre orientation
**Specific wear rate**	10^−6^–10^−5^ mm^3^/(N·m)	For gears

**Table 2:** Comparison of representative CFRP matrix systems in terms of thermal stability, mechanical performance, chemical resistance, recyclability, and processing requirements, synthesized from published experimental and review studies [9,22,30,38,43,48].

System	Tg (°C)	Maximum Service Temperature (°C)	Tensile Strength (MPa)	Chemical Resistance	Recyclability	Processing Temperature (°C)
**CF/epoxy**	120–150	~130	1000–1800	Moderate	No	120–180
**CF/BMI**	250–320	~270	1200–1600	Good	No	180–230
**CF/PEEK**	~143	~250	1400–1600	Excellent	Yes	360–400
**CF/PPS**	85–100	~240	700–900	Excellent	Yes	300–340
**CF/PEKK**	156–175	~220	900–1300	Excellent	Yes	340–380
**CF/vitrimer**	80–150	~150	700–1000	Moderate–good	Yes (chemical)	150–220

**Table 3:** Functional classification of CFRP automotive powertrain components according to loading conditions, recommended matrix systems, and principal engineering limitations, synthesized from representative studies on automotive composite applications and structural design [29,30,31,34].

Category	Typical Components	Primary Loading	Recommended Matrices	Main Limitation
**4.1 Load-bearing**	Connecting rod, crankshaft	Fatigue, tension/compression	CF/epoxy, CF/PEEK	Joints, orthotropy
**4.2 Sliding/tribological**	Seals, sliding bearings	Friction, wear	CF/PEEK, CF/PPS	Fibre abrasiveness
**4.3 Rotating**	Drive shaft, gears	Torsion, fatigue, contact loading	CF/epoxy, CF/PEEK	Critical speed, pitting
**4.4 Housing/protective**	Valve cover, transmission housing	Creep, heat, sealing	CF/PA66, CF/PPS	Warpage, inserts
**4.5 Fluid-handling**	Intake manifold, oil pan	Fluid pressure, creep	CF/PA66, CF/PPS	Sealing integrity, moisture absorption

**Table 4:** Design optimization strategies for CFRP automotive powertrain components based on published studies addressing fibre orientation, lightweight structural optimization, manufacturing design, dynamic performance, and hybrid multi-material structures [9,29,30,34,62,63].

Strategy	Applicable Components	Primary Effect	Limitations
**Fibre orientation**	Shafts, gears, connecting rods	Maximization of mechanical properties in the loading direction	Manufacturing complexity, anisotropy
**Thickness gradation**	All component types	Weight reduction of 15–25%	More demanding design and manufacturing
**Ribbing**	Housings, manifolds, oil pans	Increased stiffness without significant weight increase	Fibre orientation within ribs
**Hybrid metal–CFRP structures**	Shafts, gears, housings	Utilization of the advantages of both materials	CFRP–metal joints, thermal expansion mismatch

**Table 5:** Representative operating conditions, recommended CFRP material systems, and typical mechanical properties for selected automotive powertrain components, synthesized from published experimental and review studies on high-performance CFRP composites and their engineering applications [9,29,30,31,34,64].

Component	Typical Operating Temperature (°C)	Dominant Loading Condition	Recommended CFRP System	Typical Tensile Strength (MPa)	Typical Modulus (GPa)
**Driveshaft**	80–140	Torsion, high-cycle fatigue, vibration	CF/PEEK, CF/epoxy	1200–1600	120–180
**Gear wheel**	90–180	Contact stress, pitting, wear	Hybrid steel/CF-PEEK, CF/PPS	700–1400	70–140
**Connecting rod**	120–200	Alternating tension/compression fatigue	CF/PEEK, CF/epoxy	1400–1800	130–180
**Crankshaft**	120–220	Combined bending and torsion	Hybrid metal/CFRP	1200–1600	120–170
**Valve cover**	80–140	Creep, thermal cycling, vibrations	CF/PPS, CF/PA66	150–350	15–45
**Transmission housing**	90–160	Vibrations, chemical exposure, creep	CF/PPS, CF/PEEK	200–500	20–55
**Oil pan**	90–150	Fluid pressure, chemical exposure	CF/PPS, CF/PA66	180–400	15–40
**Intake manifold**	80–180	Pressure pulsation, vibrations	CF/PA66, CF/PPS	180–450	15–50
**Sliding bearings/bushings**	100–220	Friction, wear, contact fatigue	CF/PEEK, CF/PPS	250–600	20–60
**Sealing components**	80–160	Pressure, creep, chemical exposure	CF/PPS, CF/PA66	150–350	15–40
**Brackets and supports**	60–140	Static loading, vibrations	CF/PA66, CF/epoxy	200–500	20–60
**Turbocharger-adjacent components**	180–260	Extreme temperature, oxidation	CF/BMI, CF/PEEK	1200–1600	120–180

Note: The reported operating temperatures and mechanical property ranges represent typical values synthesized from multiple published studies. The exact values depend on laminate architecture, fibre volume fraction, manufacturing process, specimen geometry, and testing conditions; therefore, they should be interpreted as indicative ranges rather than directly comparable material constants.

**Table 6:** Recommended fibre architectures, manufacturing technologies, dominant degradation mechanisms, and design strategies for CFRP automotive powertrain components, synthesized from representative studies on composite processing, structural design, and hybrid engineering solutions [30,31,48,58,65,66].

Component	Recommended Fiber Architecture	Preferred Manufacturing Process	Critical Degradation Mechanism	Recommended Design Strategy
**Driveshaft**	UD ±45° + 0°	Filament winding, AFP, compression molding	Delamination, creep-fatigue	Hybrid metallic joints, thin-ply, laminate optimization
**Gear wheel**	Woven + multidirectional laminate	Compression molding	Pitting, abrasive wear, interfacial damage	Metallic tooth ring, local reinforcement
**Connecting rod**	UD 0° + ± 45°	Prepreg compression molding	Fatigue cracking near pin holes	Thickness gradation, local reinforcement
**Crankshaft**	Multidirectional laminate	Hybrid compression molding	Delamination, torsional fatigue	Hybrid core design, stiffness optimization
**Valve cover**	Short fiber	Injection molding	Warping, bolt relaxation	Rib reinforcement, optimized flow orientation
**Transmission housing**	Short/long fiber	Injection molding, overmolding	Leakage, creep deformation	Metallic inserts, integrated ribs
**Oil pan**	Short fiber	Injection molding	Fluid absorption, sealing degradation	Reinforced sealing zones
**Intake manifold**	Short fiber	Injection molding	Microcracking, thermal distortion	Melt-flow optimization, rib structures
**Sliding bearings/bushings**	Short fiber	Injection molding, compression molding	Abrasive wear, pitting	PTFE/graphite additives, hybrid tribological contact
**Sealing components**	Short fiber	Injection molding	Stress relaxation, leakage	Optimized sealing surfaces
**Brackets and supports**	Short/long fiber	Injection molding	Creep, fatigue cracking	Topology optimization, rib reinforcement
**Turbocharger-adjacent components**	Continuous laminate	Hot press molding	Thermal matrix degradation	Thermal shielding, high-Tg matrices

**Table 7:** Comparison of the principal manufacturing processes for CFRP composites in terms of suitable matrix systems, fibre architecture, production cycle time, applicable automotive powertrain components, and typical void content, synthesized from representative studies on composite manufacturing technologies [30,58,61,71].

Process	Suitable Matrices	Fibre Type	Cycle Time	Suitable Components	Void Content
**Injection moulding**	PA66, PPS, PEEK	Short fibres (0.1–1 mm)	30–120 s	Housings, manifolds, oil pans	<2%
**Compression moulding (hot press)**	Epoxy, BMI, PEEK, PPS	Continuous fibres, woven fabrics	5–60 min	Gears, plates, housings	<2%
**Overmoulding**	PPS, PEEK + PA66	Continuous + short fibres	2–5 min	Hybrid housings, structural components	1–4%
**Additive manufacturing (FDM)**	PEEK, PEKK, PA	Continuous/short fibres	Hours	Prototypes, small-series parts	3–8%

**Table 8:** Engineering design framework for CFRP automotive powertrain components, summarizing recommended fibre architectures, dominant degradation mechanisms, and design strategies synthesized from representative studies on structural optimization, fatigue behaviour, and hybrid multi-material systems [29,31,34,37,62].

Component	Recommended Fibre Architecture	Critical Degradation Mechanism	Recommended Design Strategy
**Component**	UD ±45° + 0° laminate	Delamination, creep–fatigue interaction	Hybrid metallic joints, thin-ply laminates, torsional laminate optimization
**Driveshaft**	Woven multidirectional laminate	Pitting, abrasive wear, interfacial degradation	Metallic tooth ring, local reinforcement, hybrid structure
**Gear wheel**	UD 0° + ±45° laminate	Fatigue cracking near pin holes	Thickness gradation, local reinforcement near stress concentrators
**Connecting rod**	Multidirectional laminate	Delamination, torsional fatigue	Hybrid core design, stiffness optimization
**Crankshaft**	Short-fibre-reinforced composite	Warping, bolt relaxation	Rib reinforcement, optimized melt-flow orientation
**Valve cover**	Short-/long-fibre composite	Leakage, creep deformation	Metallic inserts, integrated rib structures
**Transmission housing**	Short-fibre-reinforced composite	Fluid absorption, sealing degradation	Reinforced sealing zones, ribbed structure
**Oil pan**	Short-fibre-reinforced composite	Microcracking, thermal distortion	Melt-flow optimization, integrated stiffening ribs
**Intake manifold**	Short-fibre tribological composite	Abrasive wear, contact fatigue, pitting	PTFE/graphite additives, hybrid tribological contact
**Sliding bearings/bushings**	Short-fibre-reinforced composite	Stress relaxation, leakage	Optimized sealing surfaces, local reinforcement
**Sealing components**	Short-/long-fibre composite	Creep deformation, fatigue cracking	Topology optimization, rib reinforcement
**Brackets and supports**	Continuous-fibre laminate	Thermal matrix degradation	Thermal shielding, high-Tg matrices

**Table 9:** Principal degradation mechanisms of CFRP composites in automotive powertrain applications, including operating conditions, critical structural regions, and recommended prevention strategies, synthesized from representative studies on thermomechanical behaviour, fatigue, tribological performance, and structural durability [31,34,37,38,72].

Mechanism	Conditions	Critical Regions	Prevention Strategies
**Creep failure**	Sustained loading + T > 0.8 Tg	Matrix in transverse plies	40 °C Tg margin, semi-crystalline matrices
**Fatigue cracking**	Cyclic loading, 10^7^–10^9^ cycles	90° plies, fibre–matrix interface	GNP/CNT additives, thin-ply laminates
**Delamination failure**	Thermal cycling, geometric discontinuities	Free edges, holes, bends	Interleaving, thin-ply laminates, stacking sequence optimization
**Wear/pitting**	Tribological contact, cyclic contact	Contact surface, subsurface regions	Lubrication, transfer film, hybrid structures

**Table 10:** Integrated comparison of representative CFRP material systems in terms of thermal stability, mechanical performance, chemical resistance, creep and fatigue behaviour, recyclability, and suitability for automotive powertrain temperature zones, synthesized from published experimental and review studies [22,30,38,43,48].

Material	Tg (°C)	Max. Temperature (°C)	Strength (MPa)	Chemical Resistance	Creep Resistance	Fatigue Resistance	Recyclability	Cost	Zone
**CF/epoxy**	120–150	~130	1000–1800	○	○	✓✓	✗	✓✓	1
**CF/BMI**	250–320	~270	1200–1600	✓	✓	✓✓	✗	✓	3
**CF/PEEK**	~143	~250	1400–1600	✓✓	✓✓	✓✓	✓	✗	2
**CF/PPS**	85–100	~240	700–900	✓✓	✓	✓	✓	✓	2
**CF/PEKK**	156–175	~220	900–1300	✓✓	✓✓	✓✓	✓	○	2
**CF/vitrimer**	80–150	~150	700–1000	○	○	✓	✓✓	○	1–2

Note: The symbols represent a qualitative assessment of material performance based on data reported in the literature: ✗ = poor, ○ = moderate, ✓ = good, and ✓✓ = excellent.

**Table 11:** Comparison of the principal manufacturing technologies for CFRP composites in terms of matrix system, fibre architecture, laminate quality, mechanical performance, advantages, and technological limitations, synthesized from representative studies on advanced composite manufacturing processes [30,58,61,71].

Process	Matrix Type	Fibre Type	Quality (Void Content)	Mechanical Properties	Advantages	Limitations
**Injection moulding**	PA66, PPS, PEEK	Short (<1 mm)	<2%	Lower (quasi-isotropic)	Fast production, complex geometries	Lower strength compared with continuous-fibre systems
**Compression moulding**	Epoxy, PEEK, PPS	Continuous fibres, woven fabrics	<2%	High (anisotropic)	High strength, low void content	Long production cycle
**Overmoulding**	PPS + PA66, PEEK + PPS	Hybrid/combined	1–4%	Locally high	Hybrid parts, functional integration	Interlayer adhesion issues
**AFP**	PEEK, PEKK	Continuous	<1%	Very high	Precise fibre orientation	High equipment cost
**Additive manufacturing (FDM)**	PEEK, PEKK, PA	Short/continuous	3–8%	Moderate	High design flexibility	Higher void content, lower fatigue resistance

## Data Availability

No new data were created or analyzed during this study. Data sharing is not applicable.
